# Supplementary material for: The Louisiana Amphibian Monitoring Program from 1997 to 2017: Results, analyses, and lessons learned
Source: PLoS One. 2021 Sep 30;16(9):e0257869. doi: 10.1371/journal.pone.0257869 (PMC8483421; doi:10.1371/journal.pone.0257869)
Supplement: S1 Table — Route name, NAAMP number, latitude and longitude of first stop. (DOCX) [file pone.0257869.s001.docx]

**Supplemental Table S1. LAMP Routes by name number and the coordinates of the of first stop.**

| Route Name | Route Number | Latitude | Longitude |
| --- | --- | --- | --- |
| Loranger | 420312 | 30.635638 | -90.389634 |
| Woodland | 420412 | 30.947100 | -90.915800 |
| McManus | 420211 | 30.782500 | -91.091100 |
| Tiger Bend | 420112 | 30.389600 | -90.986600 |
| Blond | 420512 | 30.585000 | -90.054300 |
| Cotton Valley | 420302 | 32.822100 | -93.289700 |
| Koran | 420502 | 32.404400 | -93.487700 |
| Roy | 420102 | 32.210300 | -93.150100 |
| Ada | 420202 | 32.487400 | -93.147800 |
| Rocky Branch | 420103 | 32.682400 | -92.200000 |
| Horseshoe Lake | 420204 | 32.728225 | -91.732492 |
| Tensas | 420104 | 32.374400 | -91.342610 |
| Monticello | 420504 | 32.613915 | -91.394927 |
| Bayou Funny Louis | 420507 | 31.818428 | -92.054343 |
| Mill Haven | 420404 | 32.480138 | -91.949244 |
| Boggy Womble | 420304 | 32.153486 | -91.571510 |
| Ansley | 420303 | 32.395833 | -92.693056 |
| Ossun | 420210 | 30.211464 | -92.075714 |
| Prairie Laurent | 420310 | 30.460700 | -92.057200 |
| Charenton | 420516 | 29.878200 | -91.455600 |
| Rayne | 420110 | 30.281368 | -92.315897 |
| Egan | 420410 | 30.274600 | -92.437200 |
| Brannon | 420411 | 29.941387 | -91.812323 |
| Bayou Jack | 420511 | 30.737960 | -91.980440 |
| Pickering | 420106 | 31.054379 | -93.195104 |
| Anacoco | 420306 | 31.335100 | -93.330000 |
| Bayou Sorrel | 420311 | 30.156181 | -91.330186 |
| Gramercy | 420212 | 30.075768 | -90.683724 |
| Otis | 420207 | 31.210000 | -92.807000 |
| Falgout Canal | 420317 | 29.400100 | -90.791600 |
| Palmetto | 420111 | 29.830999 | -92.943292 |
| Belle River | 420316 | 29.913304 | -91.229002 |
| Hecker | 420409 | 30.353655 | -93.082066 |
| Price Lake | 420515 | 29.687200 | -92.836000 |
| Lake Fourteen | 420315 | 29.637200 | -92.570100 |
| Holly Beach | 420314 | 29.834285 | -93.492289 |
| Le Bleu | 420209 | 30.313194 | -93.078491 |
| Headquarter Canal | 420215 | 29.704640 | -92.767816 |
| De Quincy | 420509 | 30.475395 | -93.434411 |
| Jennings | 420510 | 30.170300 | -92.688900 |
| Montegut | 420517 | 29.473700 | -90.555300 |
| Merryville | 420309 | 30.762609 | -93.542642 |
| Big Woods | 420109 | 30.260710 | -93.588639 |
| Antonia | 420407 | 31.549100 | -92.310600 |
| Choctaw | 420117 | 29.858628 | -90.677744 |
| Old Brannon | 420611 | 29.962000 | -91.783700 |
| Little Chenier | 420115 | 29.830999 | -92.943292 |
| Venice | 420418 | 29.276944 | -89.354722 |
| Boothville | 420318 | 29.343611 | -89.419722 |
| Phoenix | 420118 | 29.646111 | -89.939722 |
| Violet | 420518 | 29.901244 | -89.896860 |
| Wine Bayou | 420116 | 29.755700 | -90.903000 |
| Odra | 420307 | 31.601300 | -92.890300 |
| Old Blonde | 420612 | 30.260710 | -93.588639 |

Route name, NAAMP number, UTM coordinates of the first stop.
