# Supplementary figures and images for: The Louisiana Amphibian Monitoring Program from 1997 to 2017: Results, analyses, and lessons learned
Source: PLoS One. 2021 Sep 30;16(9):e0257869. doi: 10.1371/journal.pone.0257869 (PMC8483421; doi:10.1371/journal.pone.0257869)

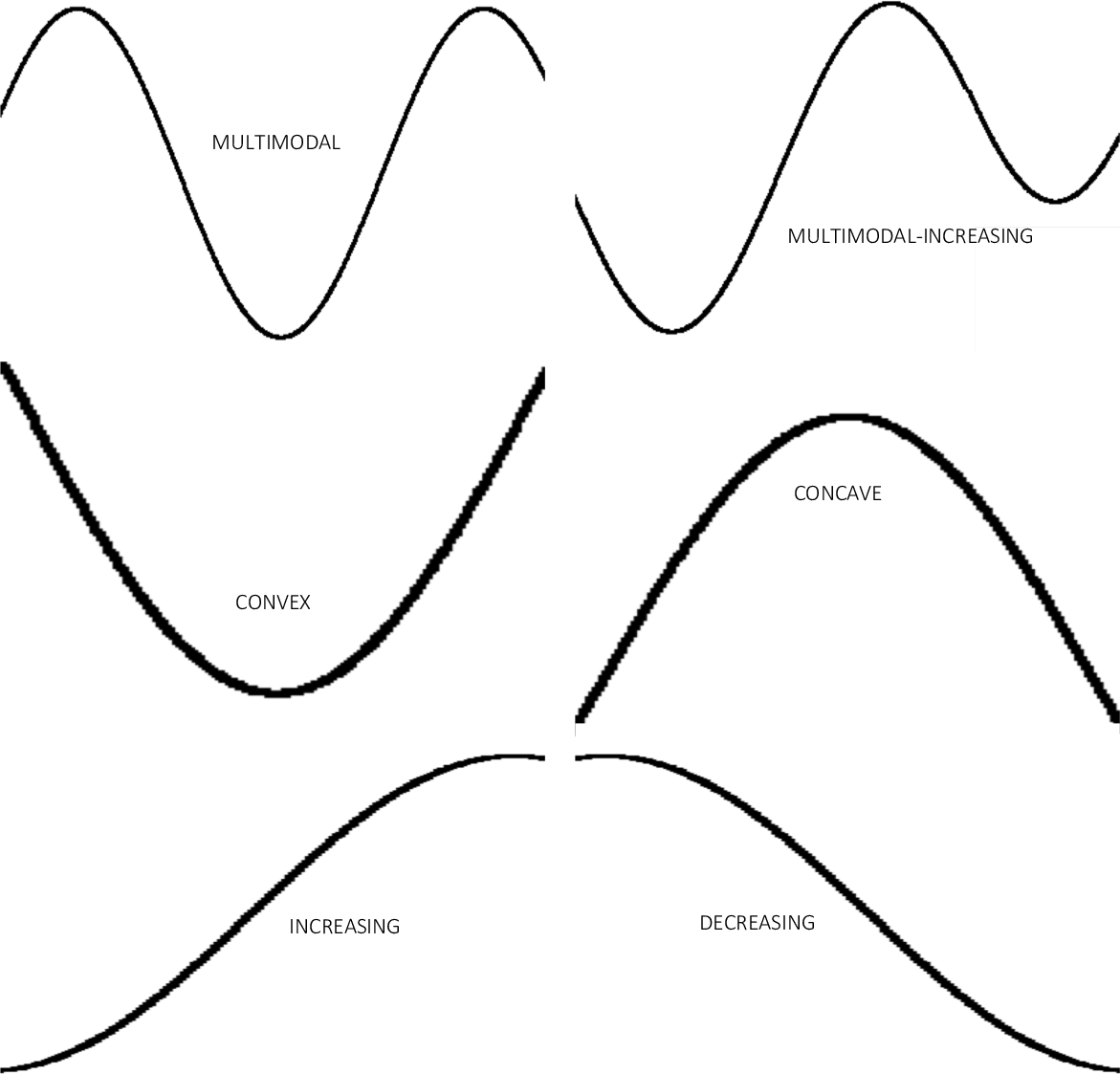

Supplement: S1 File — GAM plots of the percentage of stops a species was observed calling along a given route-run versus year. (ZIP) [file pone.0257869.s002.zip › supplental figures for PLOS/Fig1.tif]

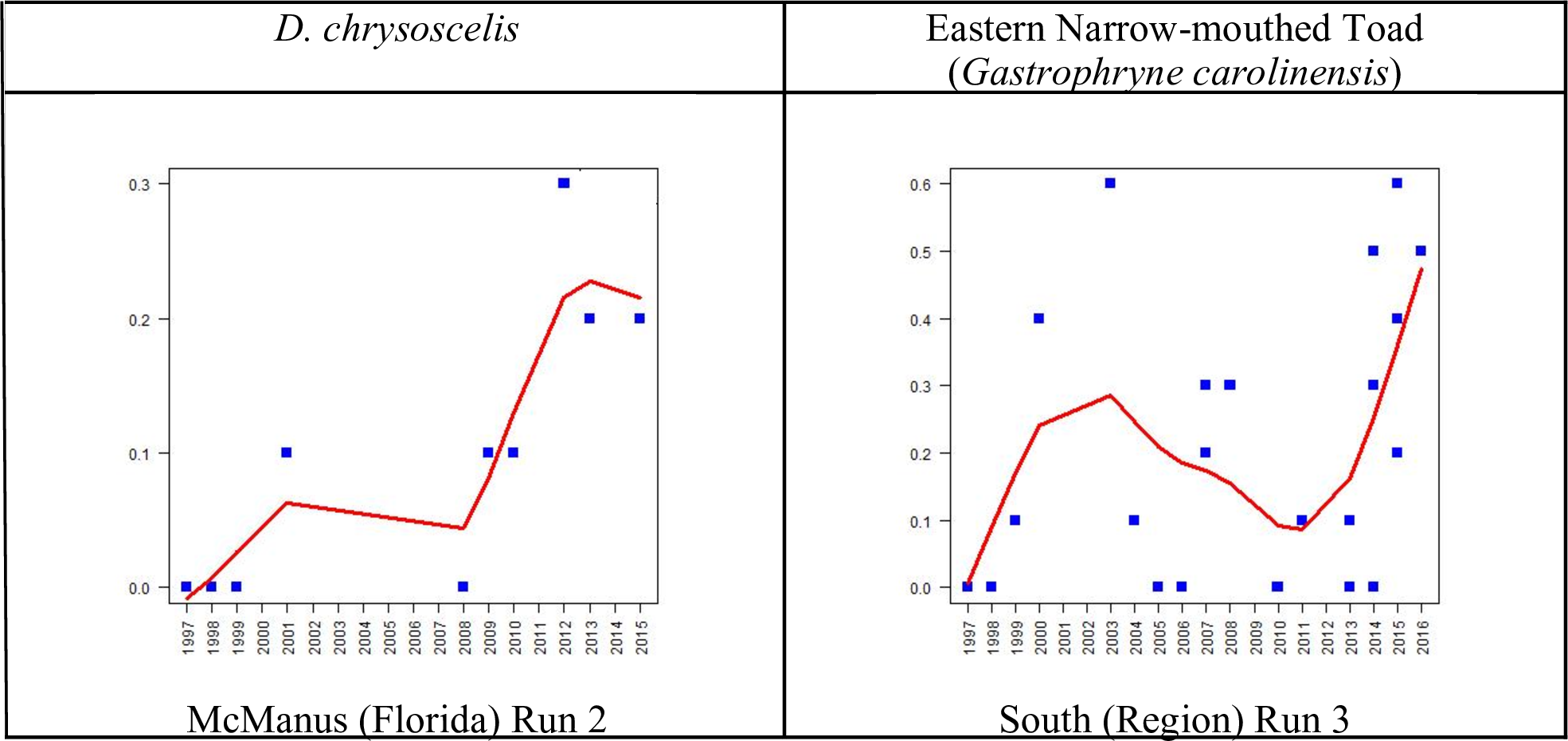

Supplement: S1 File — GAM plots of the percentage of stops a species was observed calling along a given route-run versus year. (ZIP) [file pone.0257869.s002.zip › supplental figures for PLOS/Fig10.tif]

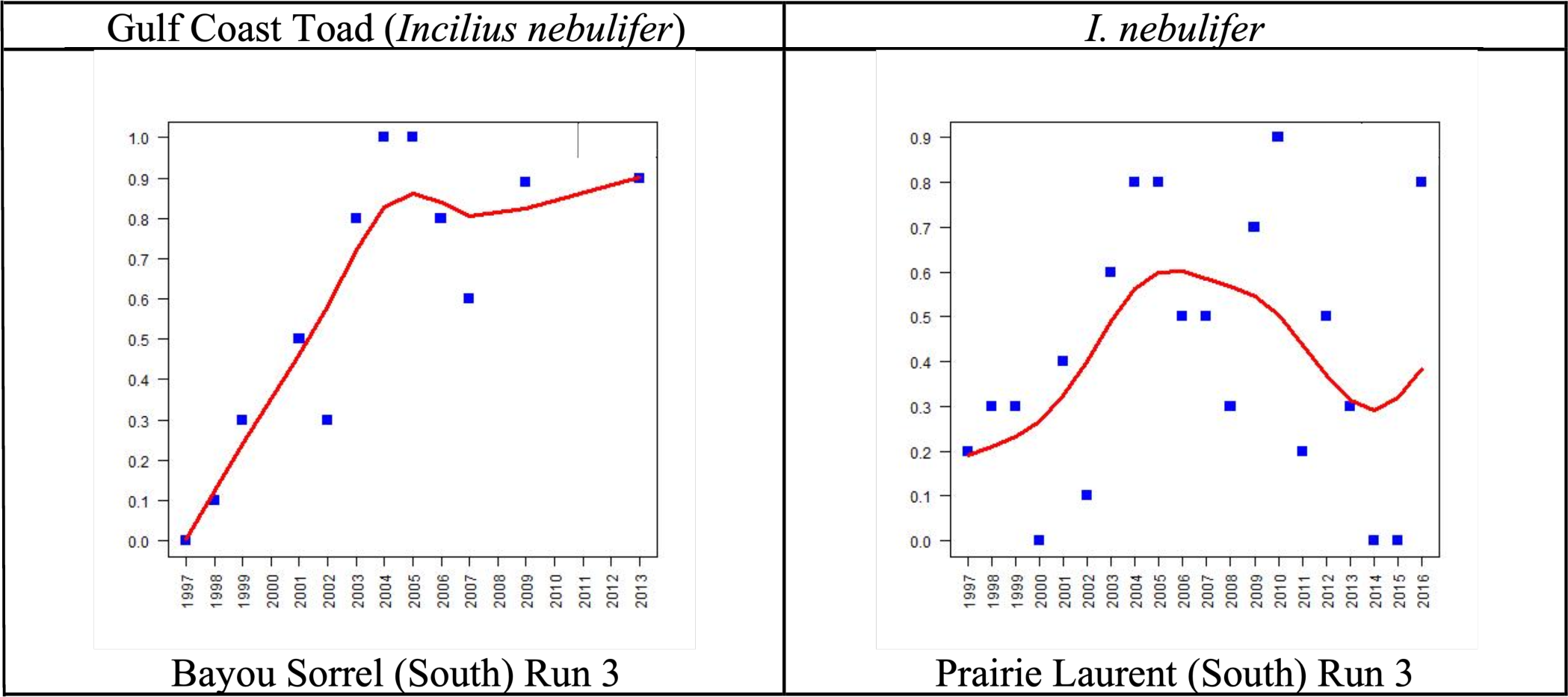

Supplement: S1 File — GAM plots of the percentage of stops a species was observed calling along a given route-run versus year. (ZIP) [file pone.0257869.s002.zip › supplental figures for PLOS/Fig11.tif]

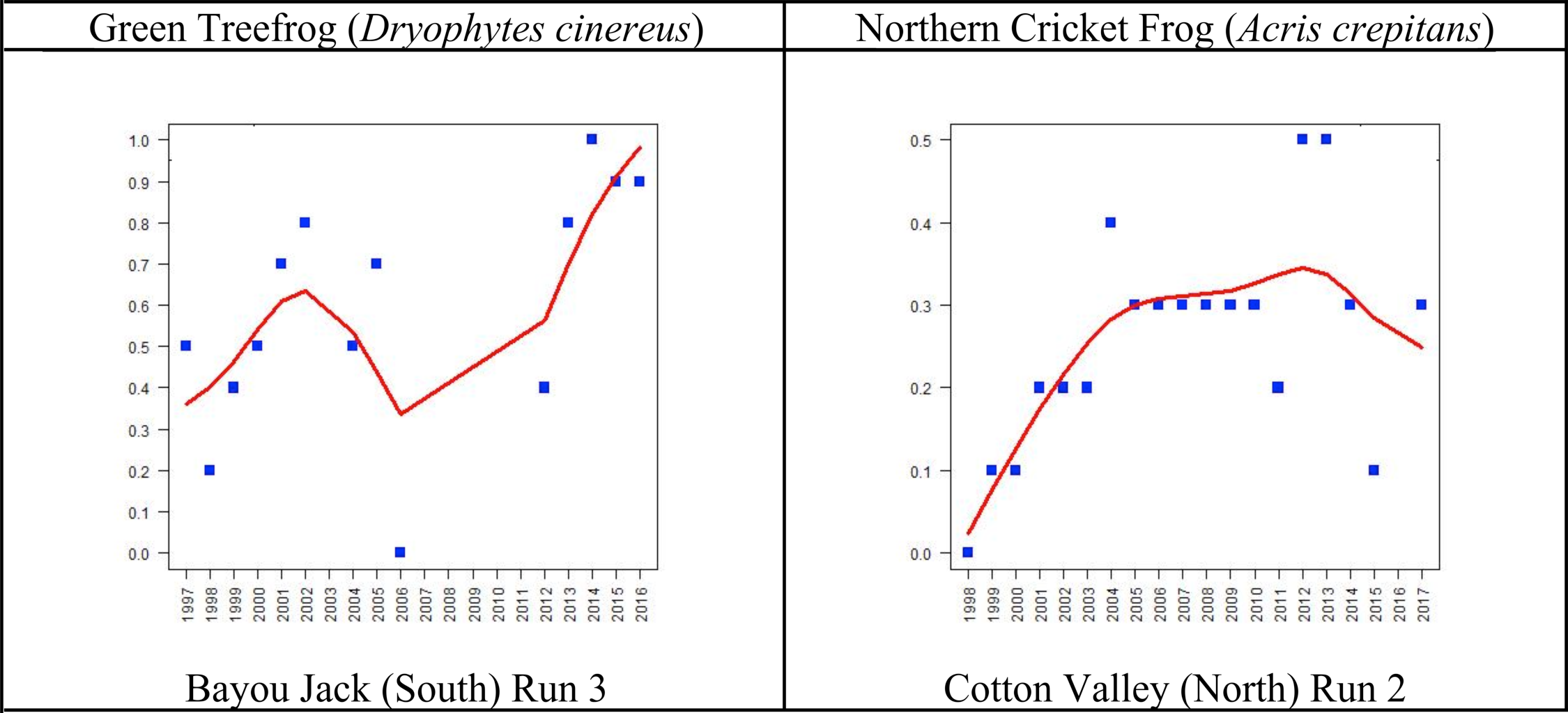

Supplement: S1 File — GAM plots of the percentage of stops a species was observed calling along a given route-run versus year. (ZIP) [file pone.0257869.s002.zip › supplental figures for PLOS/Fig12.tif]

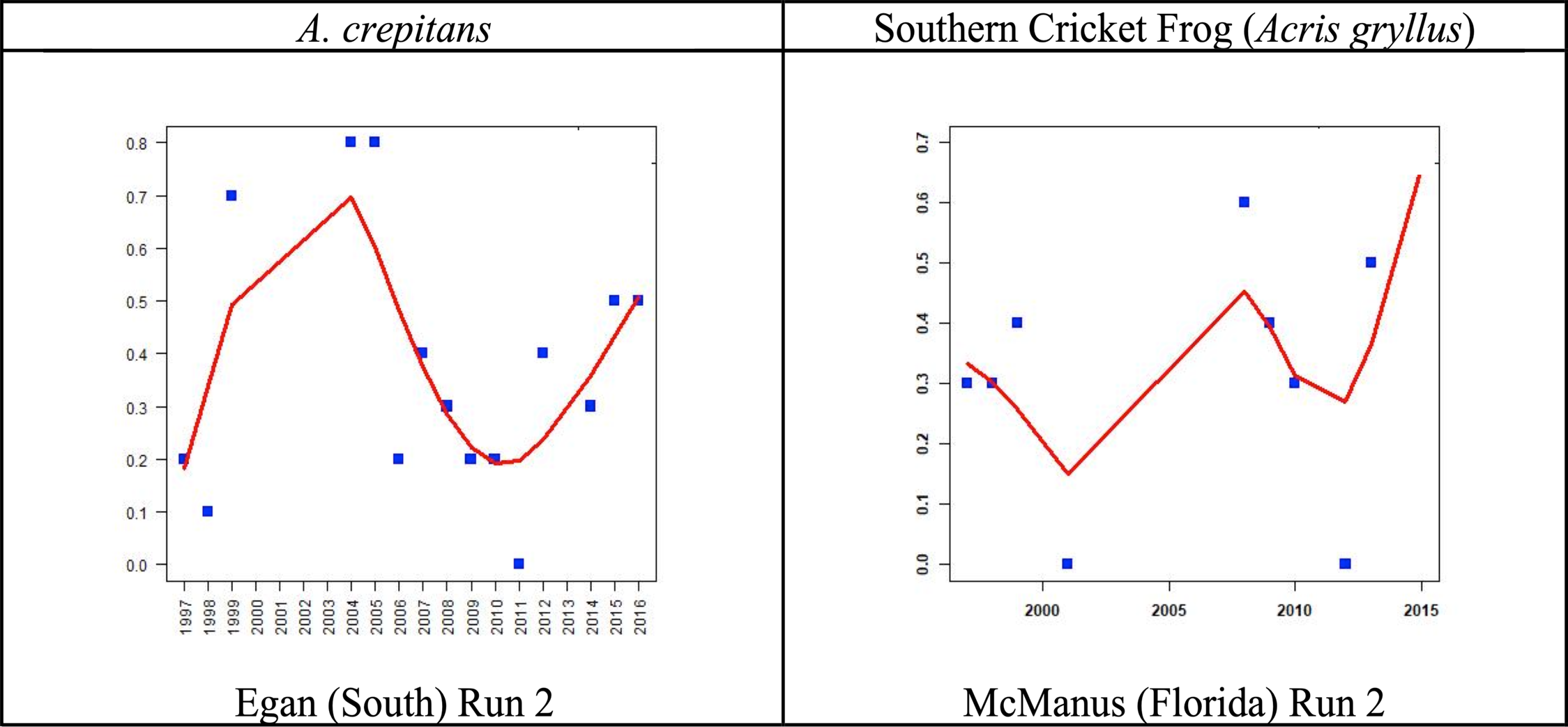

Supplement: S1 File — GAM plots of the percentage of stops a species was observed calling along a given route-run versus year. (ZIP) [file pone.0257869.s002.zip › supplental figures for PLOS/Fig13.tif]

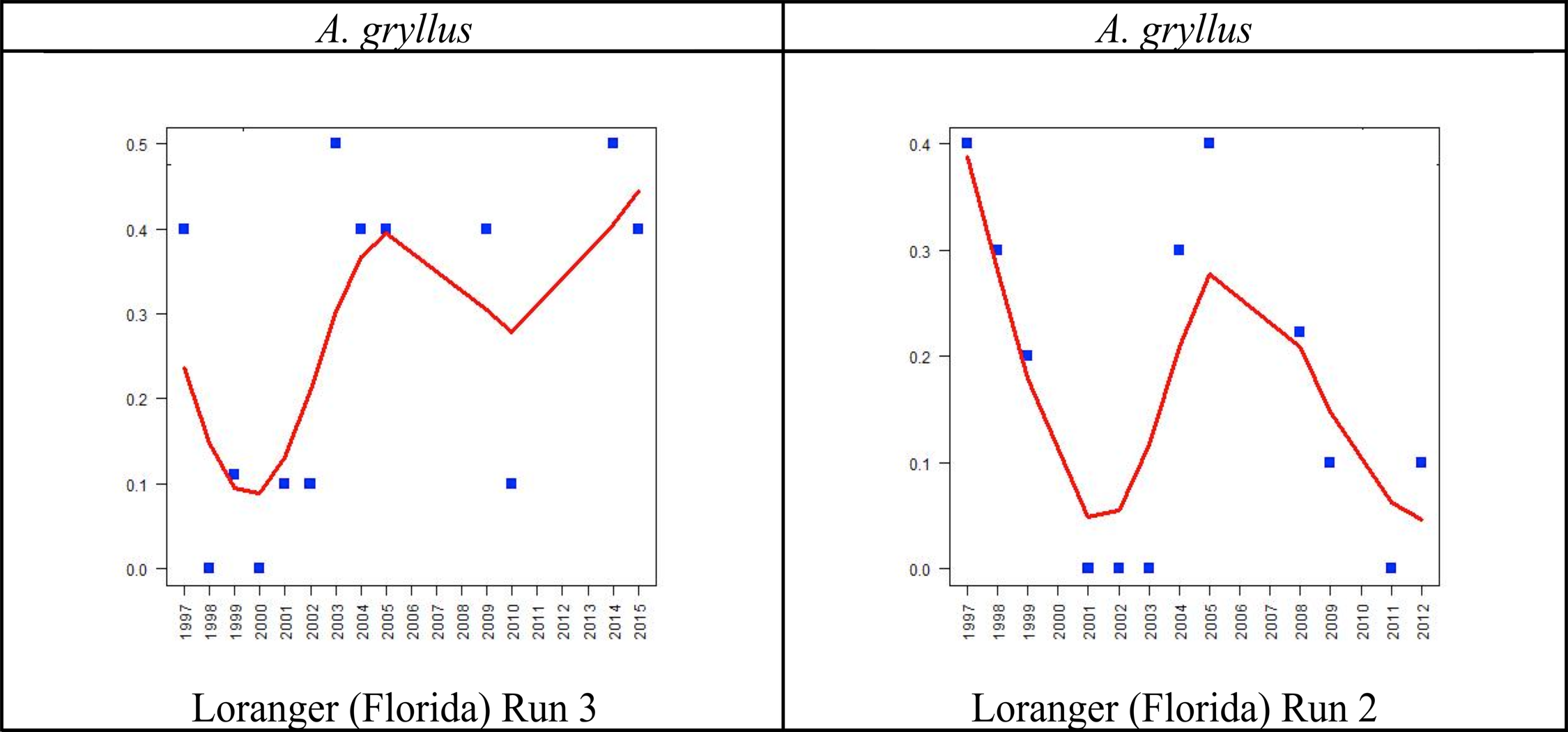

Supplement: S1 File — GAM plots of the percentage of stops a species was observed calling along a given route-run versus year. (ZIP) [file pone.0257869.s002.zip › supplental figures for PLOS/Fig14.tif]

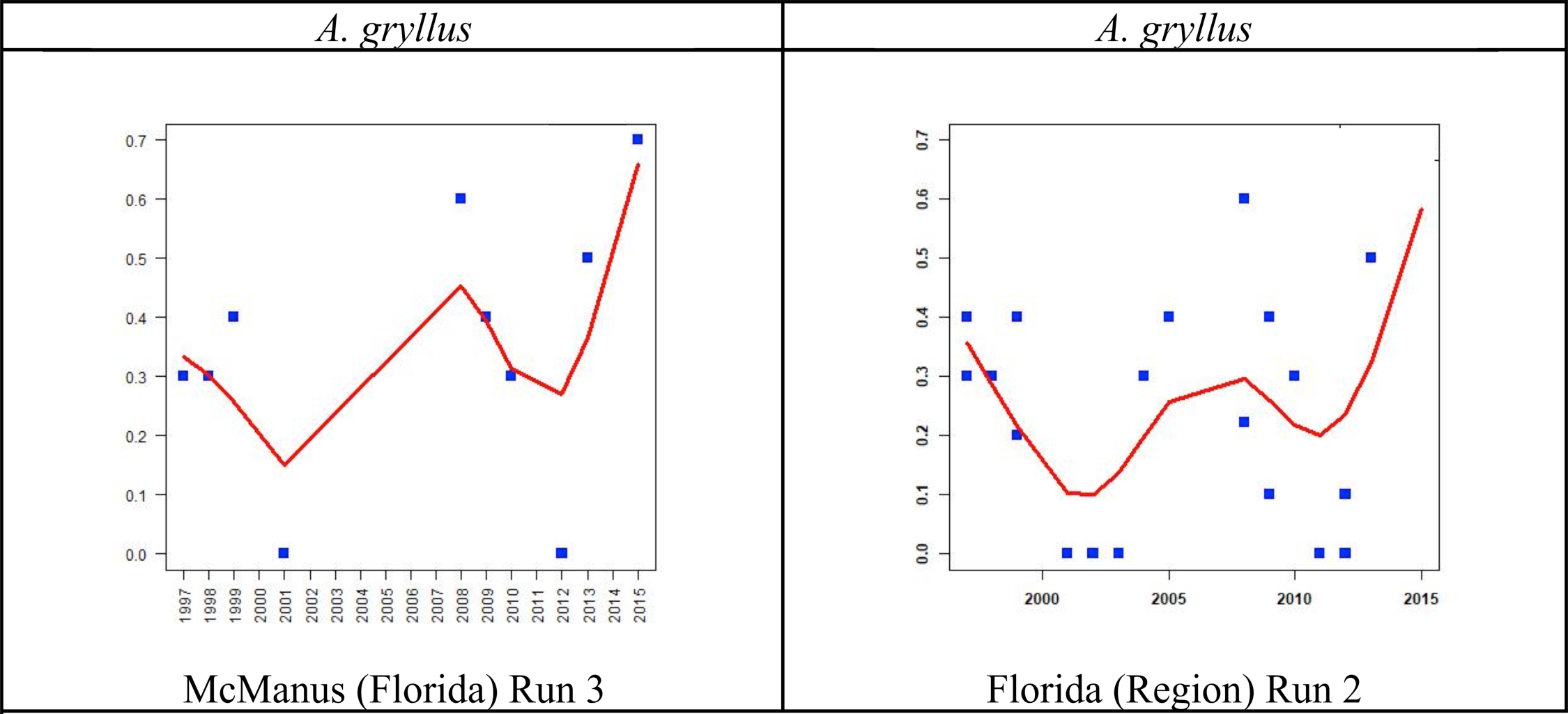

Supplement: S1 File — GAM plots of the percentage of stops a species was observed calling along a given route-run versus year. (ZIP) [file pone.0257869.s002.zip › supplental figures for PLOS/Fig15.tif]

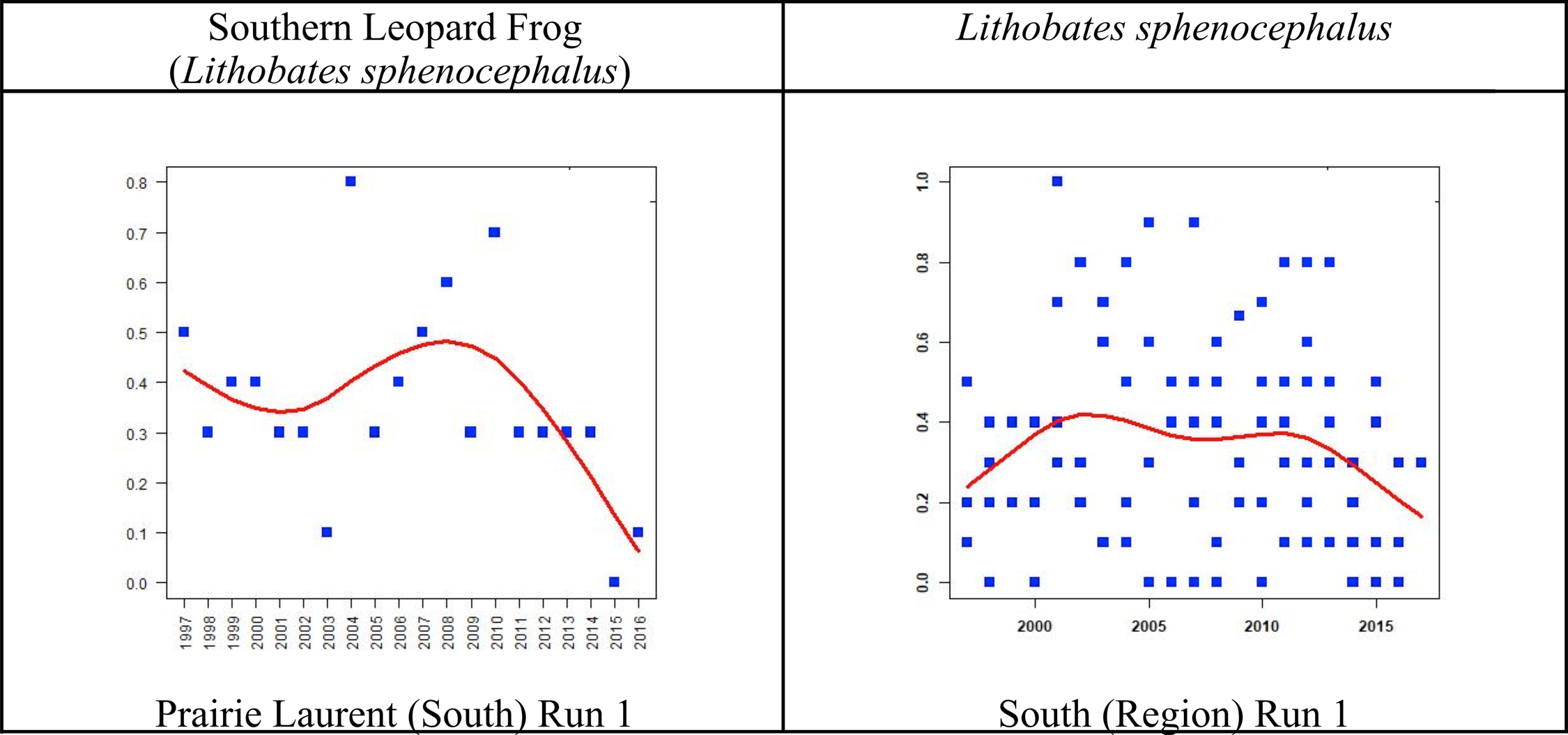

Supplement: S1 File — GAM plots of the percentage of stops a species was observed calling along a given route-run versus year. (ZIP) [file pone.0257869.s002.zip › supplental figures for PLOS/Fig16.tif]

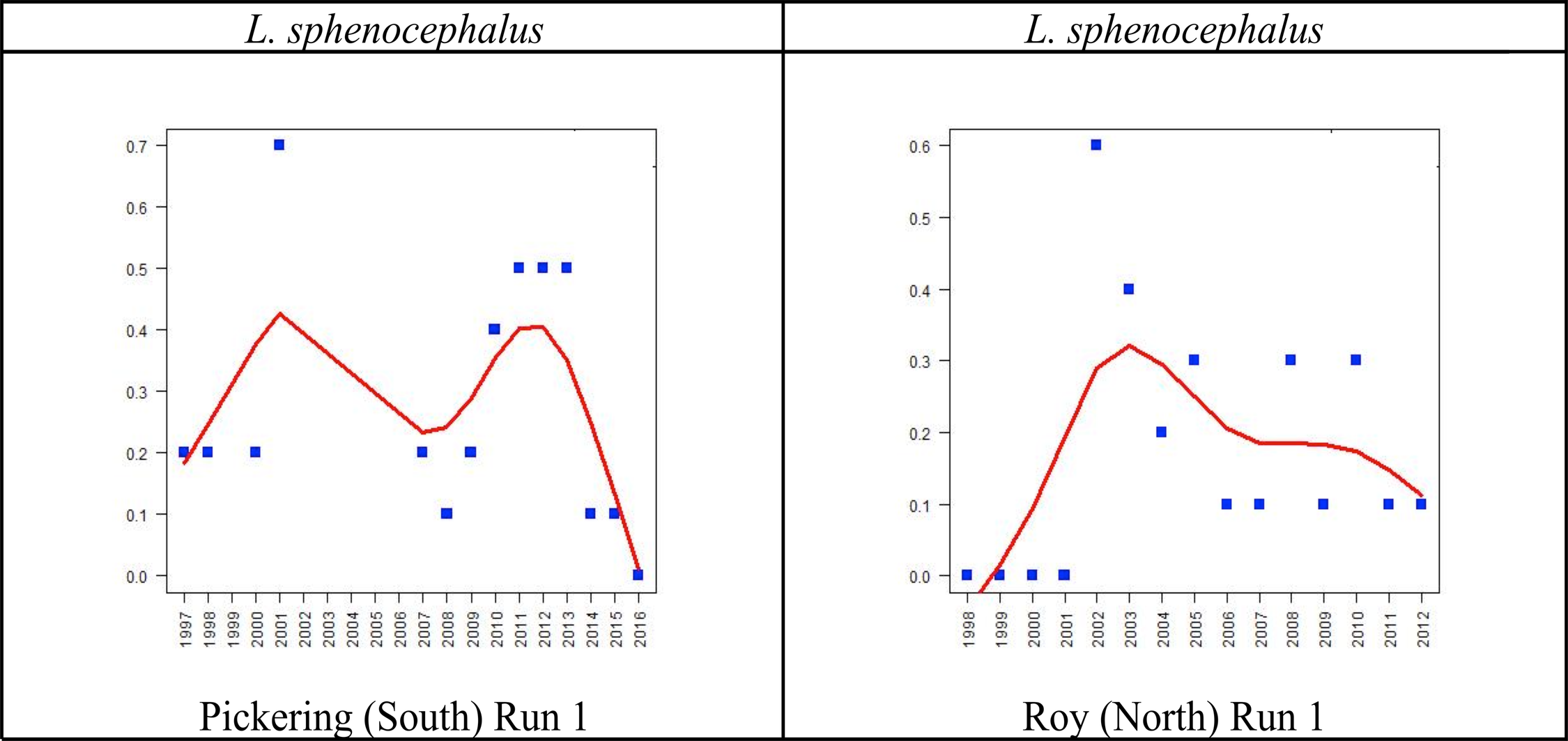

Supplement: S1 File — GAM plots of the percentage of stops a species was observed calling along a given route-run versus year. (ZIP) [file pone.0257869.s002.zip › supplental figures for PLOS/Fig17.tif]

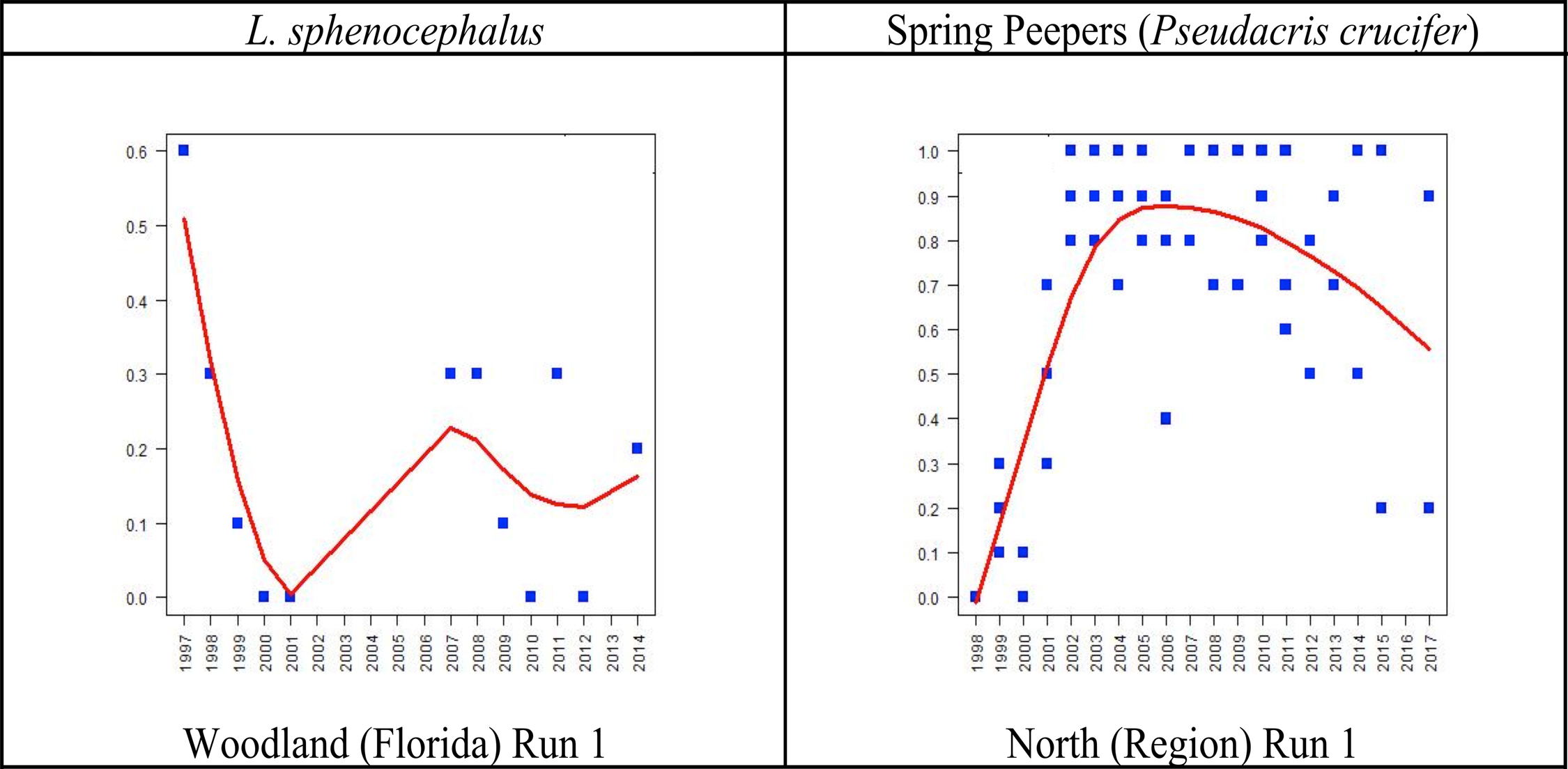

Supplement: S1 File — GAM plots of the percentage of stops a species was observed calling along a given route-run versus year. (ZIP) [file pone.0257869.s002.zip › supplental figures for PLOS/Fig18.tif]

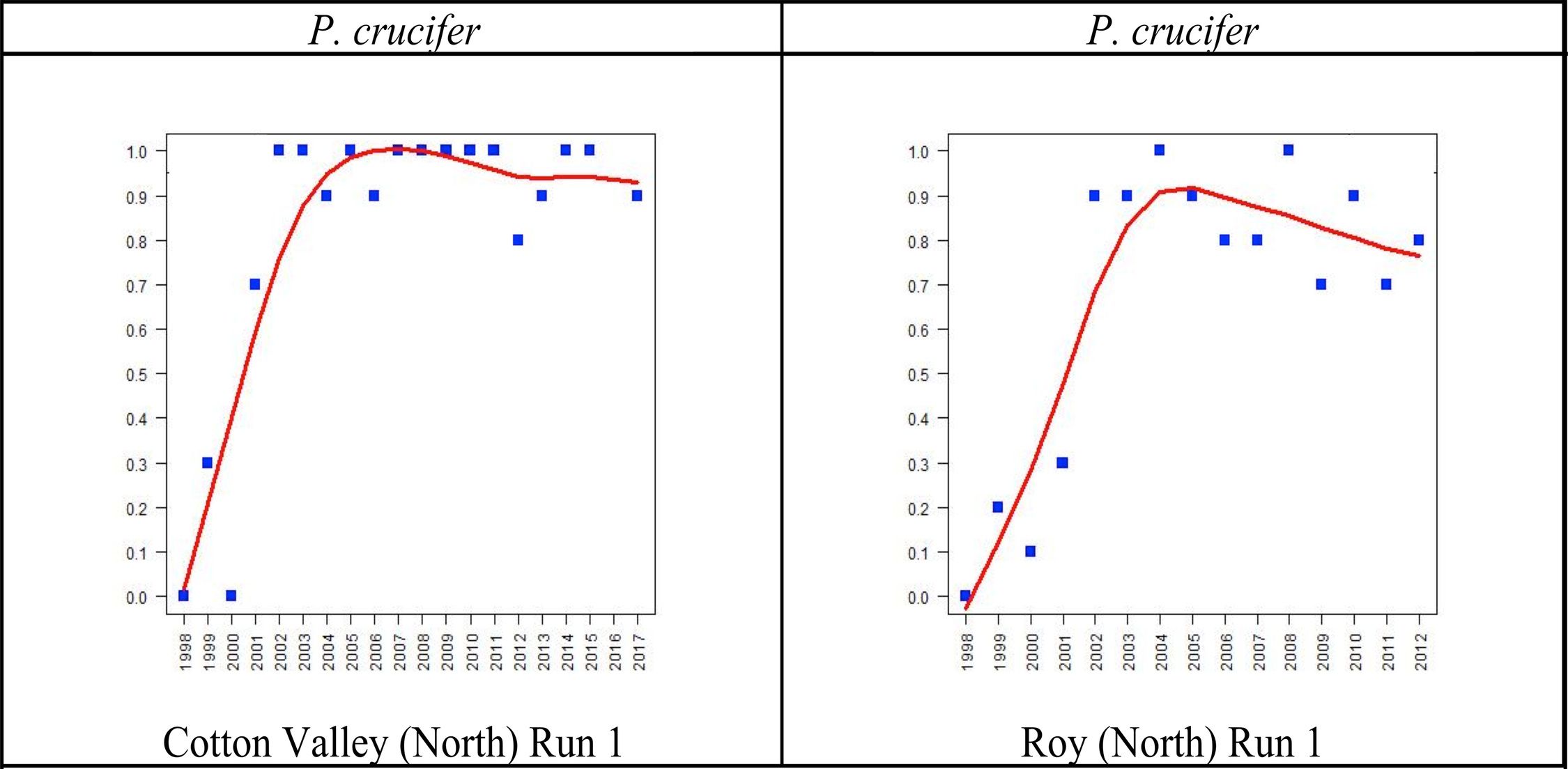

Supplement: S1 File — GAM plots of the percentage of stops a species was observed calling along a given route-run versus year. (ZIP) [file pone.0257869.s002.zip › supplental figures for PLOS/Fig19.tif]

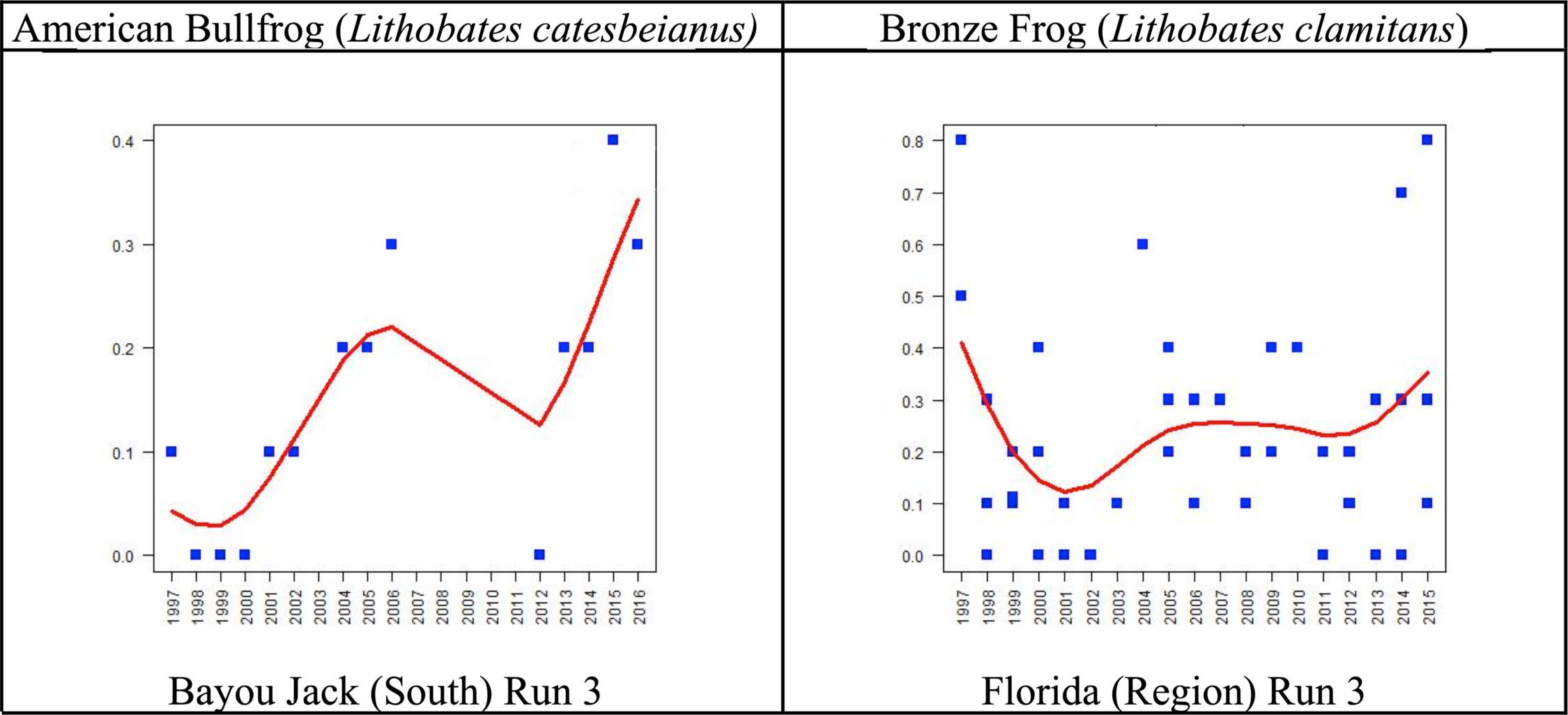

Supplement: S1 File — GAM plots of the percentage of stops a species was observed calling along a given route-run versus year. (ZIP) [file pone.0257869.s002.zip › supplental figures for PLOS/Fig2.tif]

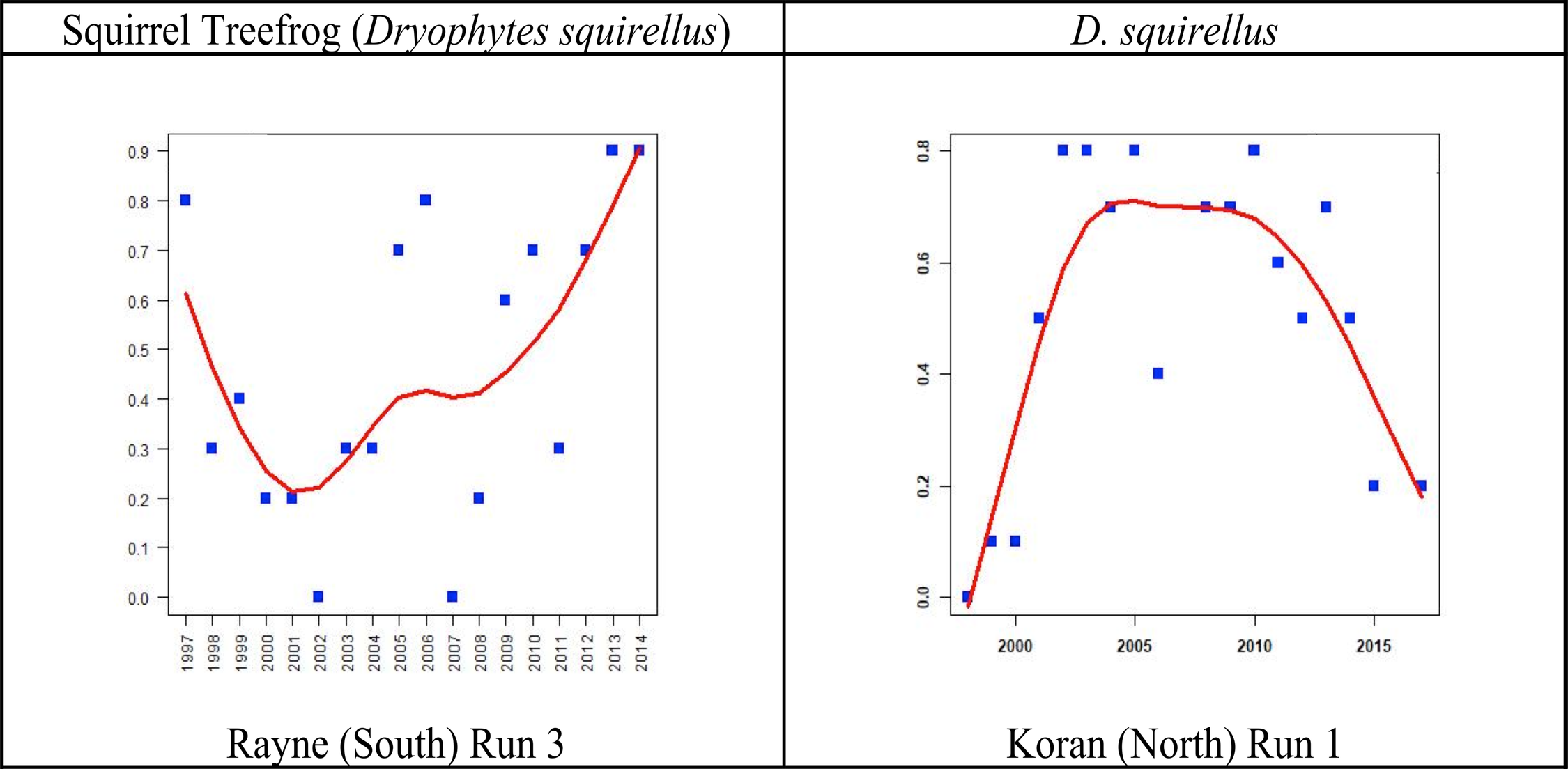

Supplement: S1 File — GAM plots of the percentage of stops a species was observed calling along a given route-run versus year. (ZIP) [file pone.0257869.s002.zip › supplental figures for PLOS/Fig20.tif]

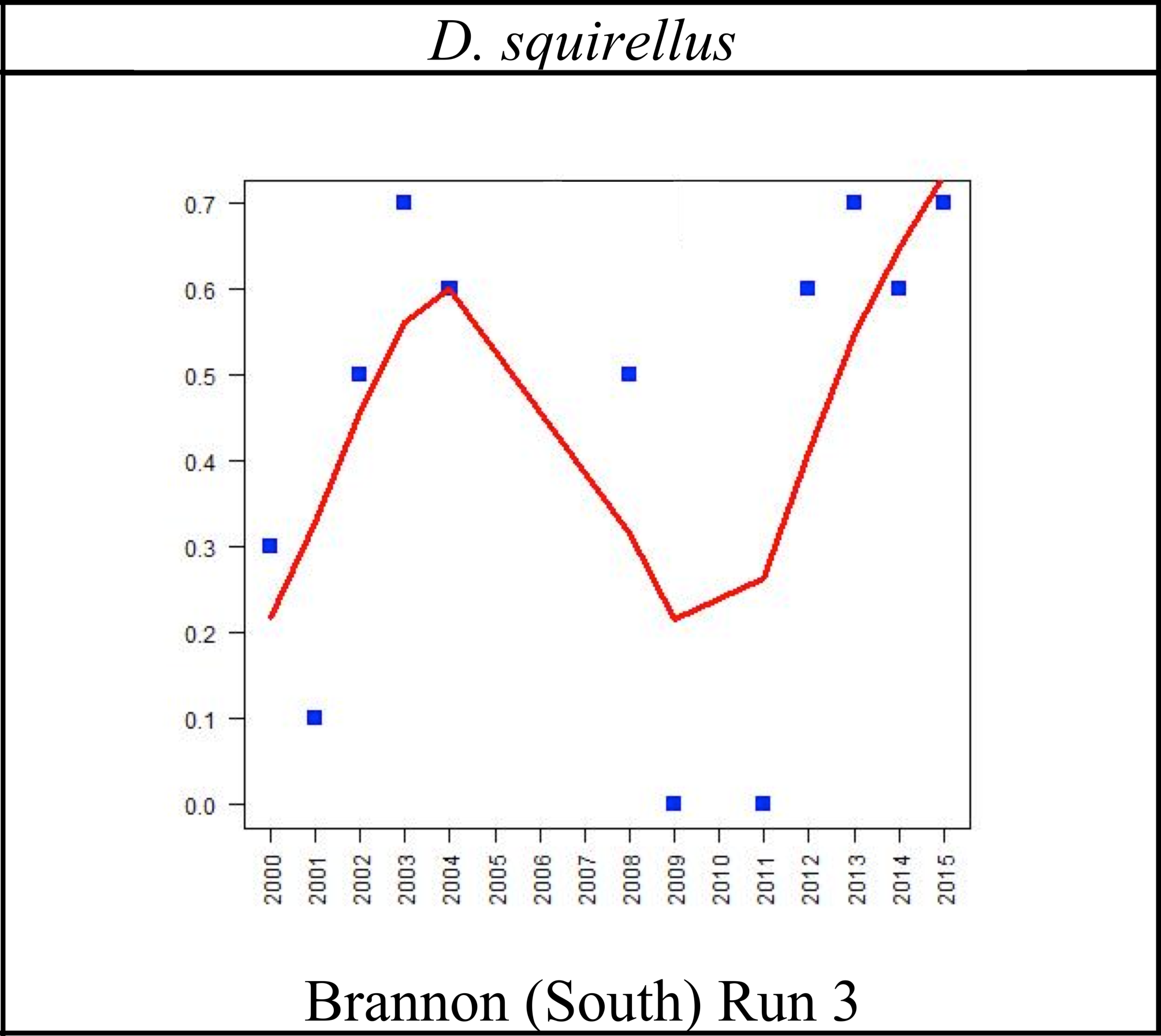

Supplement: S1 File — GAM plots of the percentage of stops a species was observed calling along a given route-run versus year. (ZIP) [file pone.0257869.s002.zip › supplental figures for PLOS/Fig21.tif]

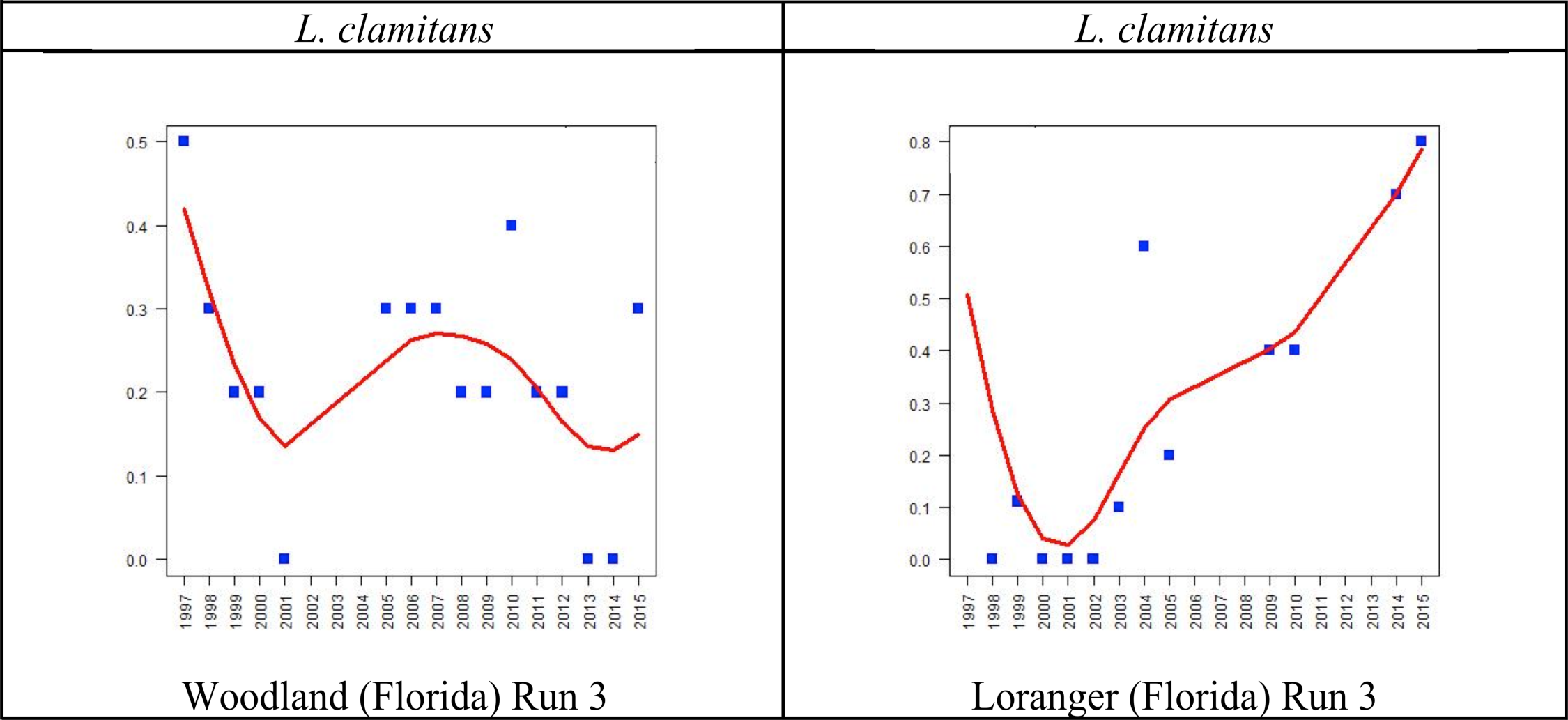

Supplement: S1 File — GAM plots of the percentage of stops a species was observed calling along a given route-run versus year. (ZIP) [file pone.0257869.s002.zip › supplental figures for PLOS/Fig3.tif]

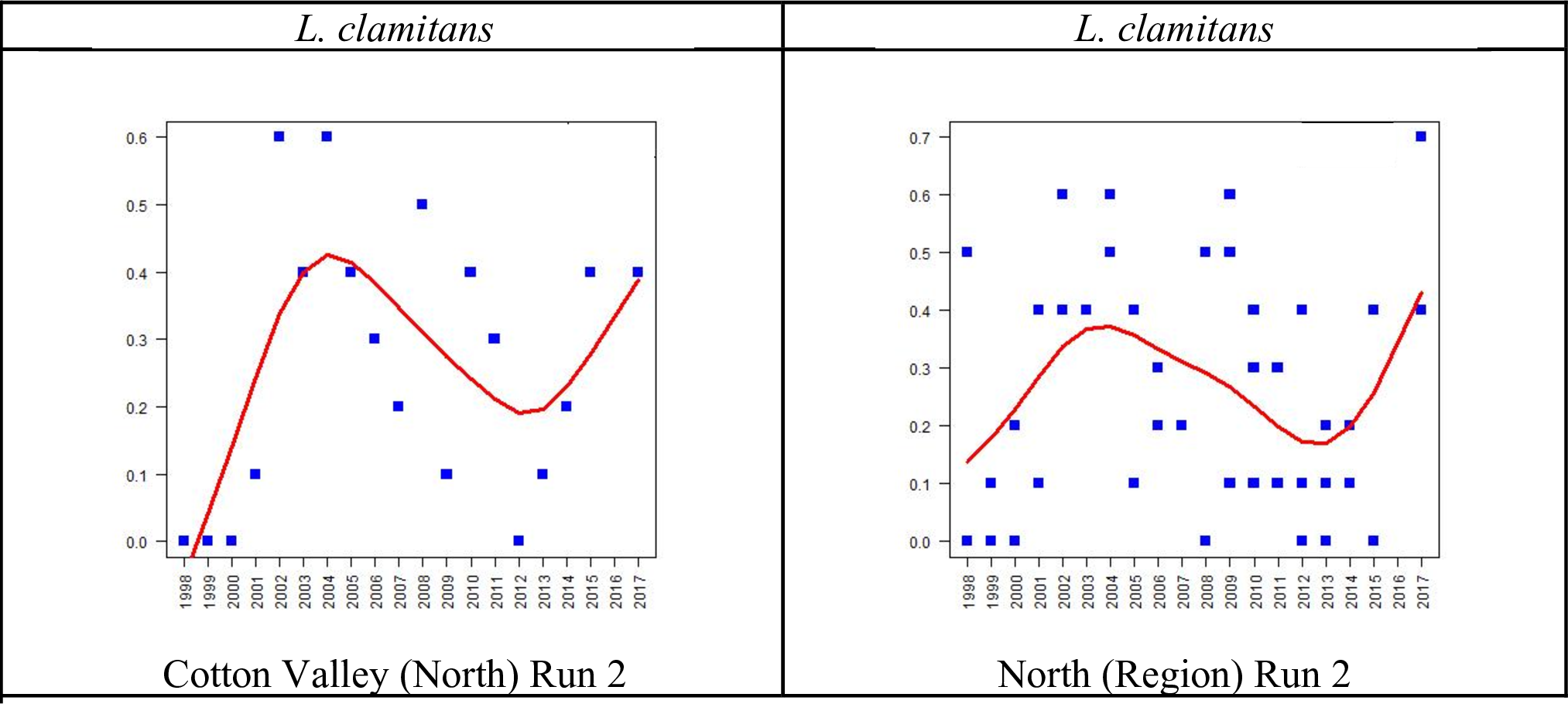

Supplement: S1 File — GAM plots of the percentage of stops a species was observed calling along a given route-run versus year. (ZIP) [file pone.0257869.s002.zip › supplental figures for PLOS/Fig4.tif]

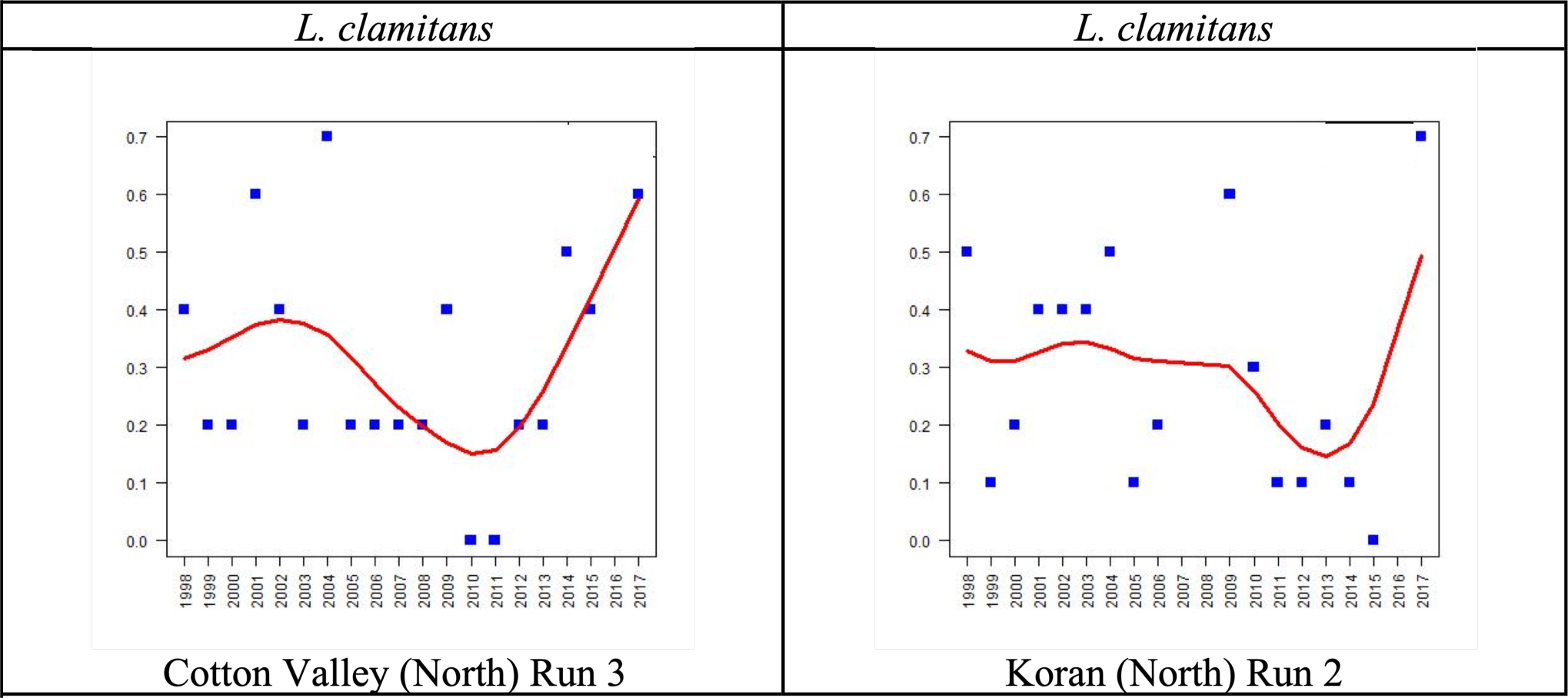

Supplement: S1 File — GAM plots of the percentage of stops a species was observed calling along a given route-run versus year. (ZIP) [file pone.0257869.s002.zip › supplental figures for PLOS/Fig5.tif]

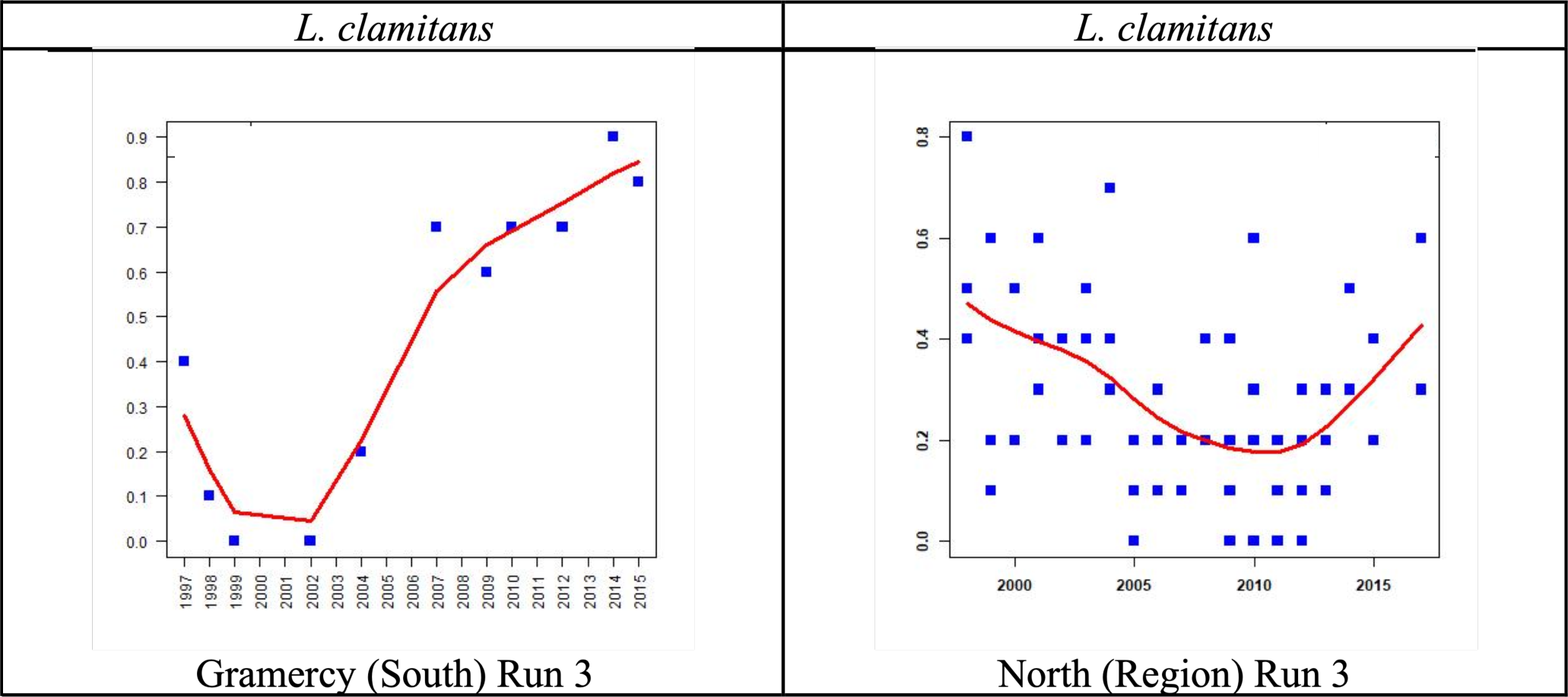

Supplement: S1 File — GAM plots of the percentage of stops a species was observed calling along a given route-run versus year. (ZIP) [file pone.0257869.s002.zip › supplental figures for PLOS/Fig6.tif]

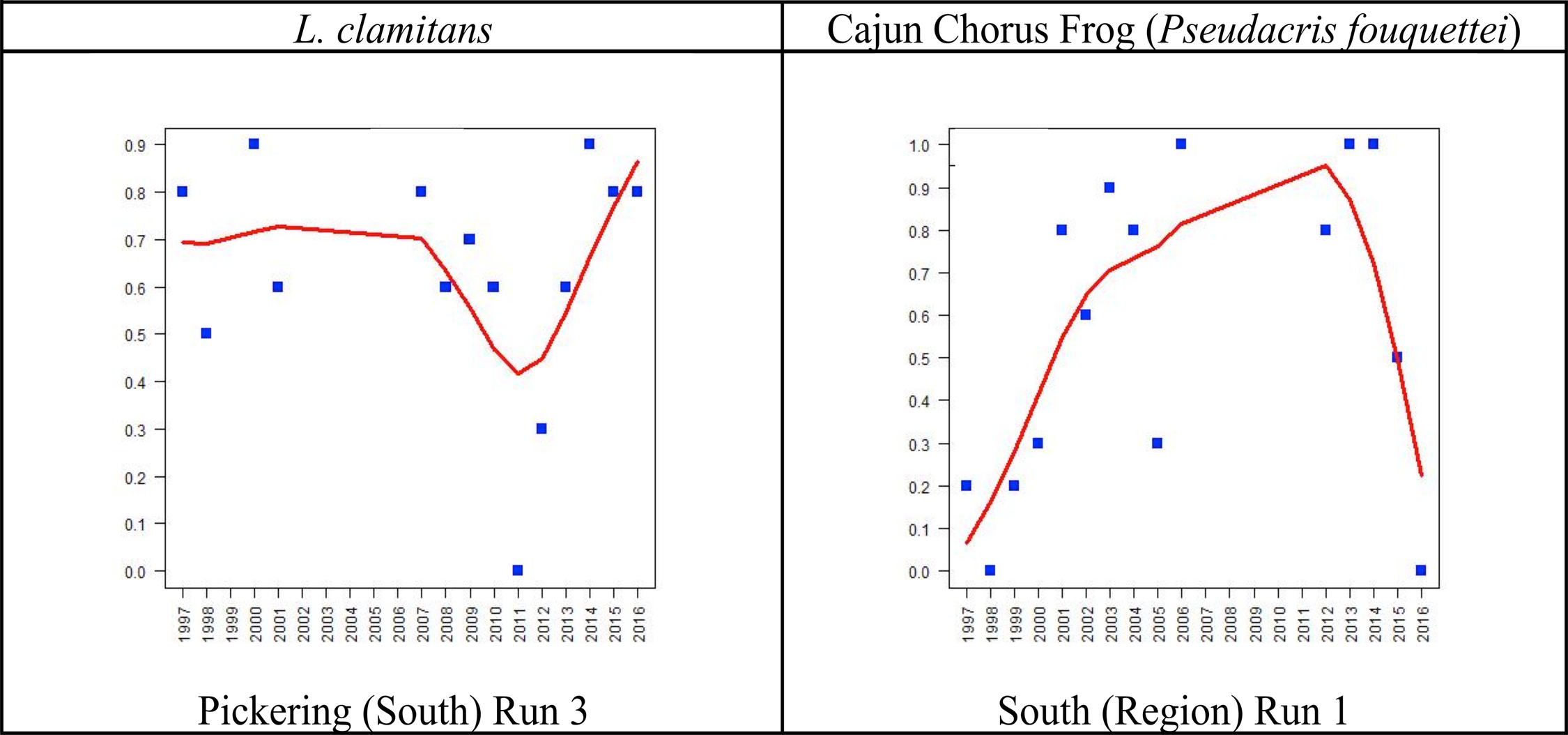

Supplement: S1 File — GAM plots of the percentage of stops a species was observed calling along a given route-run versus year. (ZIP) [file pone.0257869.s002.zip › supplental figures for PLOS/Fig7.tif]

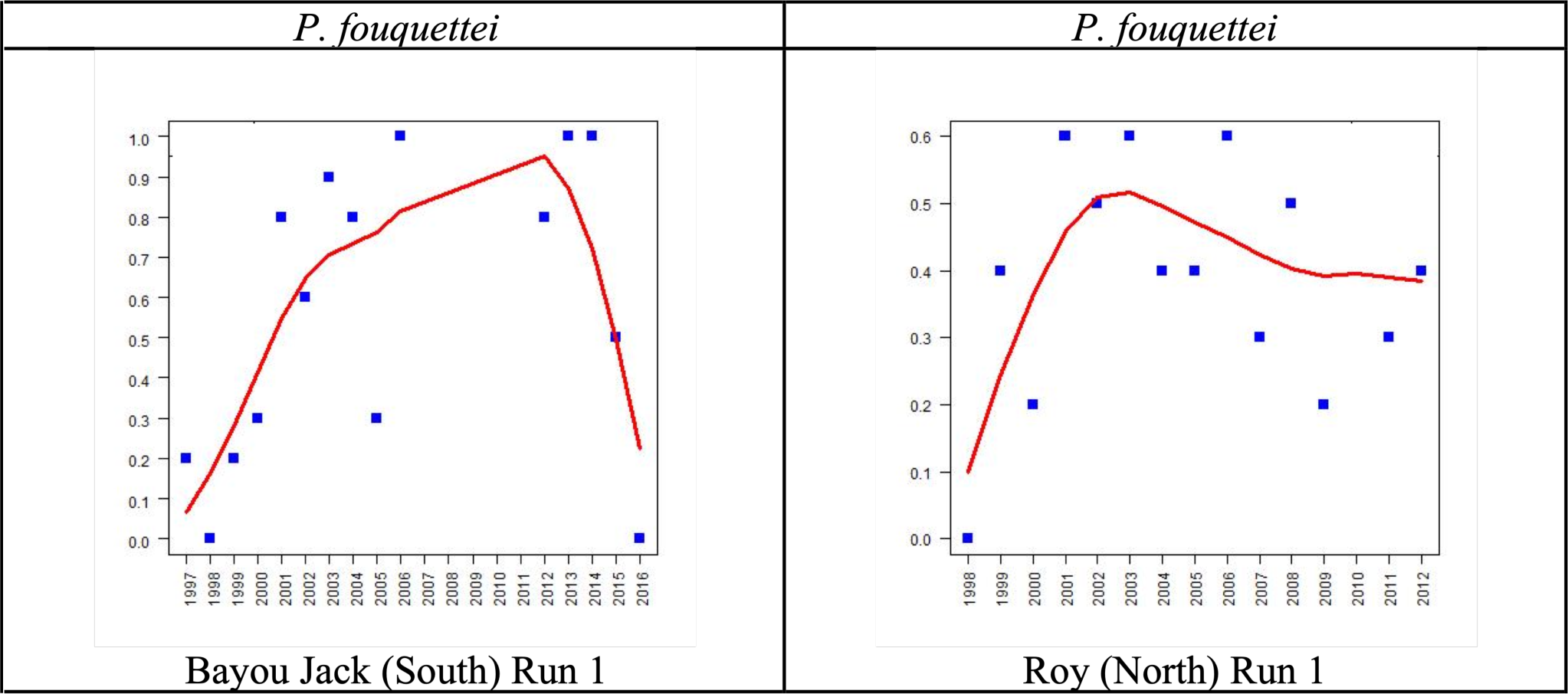

Supplement: S1 File — GAM plots of the percentage of stops a species was observed calling along a given route-run versus year. (ZIP) [file pone.0257869.s002.zip › supplental figures for PLOS/Fig8.tif]

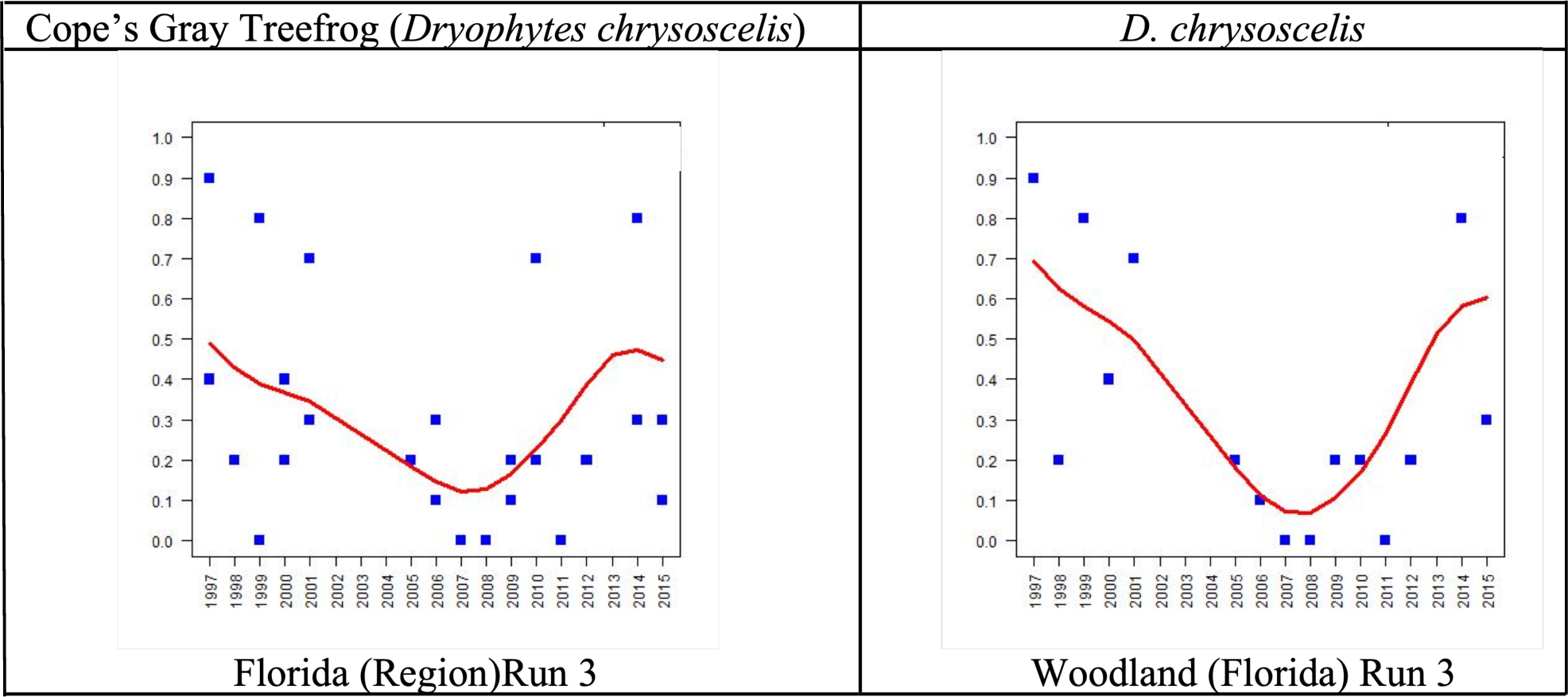

Supplement: S1 File — GAM plots of the percentage of stops a species was observed calling along a given route-run versus year. (ZIP) [file pone.0257869.s002.zip › supplental figures for PLOS/Fig9.tif]
