# Supplementary material for: The Louisiana Amphibian Monitoring Program from 1997 to 2017: Results, analyses, and lessons learned
Source: PLoS One. 2021 Sep 30;16(9):e0257869. doi: 10.1371/journal.pone.0257869 (PMC8483421; doi:10.1371/journal.pone.0257869)
Supplement: Attachment — Submitted filename: Reconciliation for PONE-D-20-21124.docx [file pone.0257869.s003.docx]

United States Department of the Interior

U.S. GEOLOGICAL SURVEY

# Jacoby Carter, Ph.D.

Research Ecologist

Wetland and Aquatic Research Center

700 Cajundome Boulevard

Lafayette, Louisiana 70506

Cell: 3370781-4882

Phone: 337-266-8620

Fax: 337-266-8586

e-mail: jacoby_carter@usgs.gov

To:	Yong Zhang, Academic Editor, PLOS ONE

Date:	Sunday, January 31, 2021

Re:	PONE-D-20-21124 Manuscript submission. Reconciliation and Response to Reviewers.

Dear Dr. Zhang,

I found the comments from the reviewers very helpful and made most of the changes they suggested (see below). In addition to the comments from your reviewers I also got some additional input by my coauthors and from the USGS review process. The number of edits and changes were so many that the mark up copy with all the track changes was a mess, with changes by one coauthor revised by a second, and so on. I merged the original document I submitted to the revised version. The resulting document was the revised document with all the changes from the original document tracked. All changes are credited to me. I have started the process of placing the data we used on ScienceBase, the USGS online database. I have been assigned a digital object identifier and the data should be available online soon.

We have concluded our internal review process and the manuscript has been approved for publication your journal.

**Comments from the editor.**

1. Please ensure that your manuscript meets PLOS ONE's style requirements, including those for file naming. The PLOS ONE style templates can be found at

https://journals.plos.org/plosone/s/file?id=wjVg/PLOSOne_formatting_sample_main_body.pdf and

https://journals.plos.org/plosone/s/file?id=ba62/PLOSOne_formatting_sample_title_authors_affiliations.pdf

Response: We went through the manuscript using the examples provided. We think it is in compliance with your style requirements.

2. In your Methods section, please provide additional location information of the study sites, including geographic coordinates for the data set if available.

Response We added the following sentence to the methods section ‘Survey Routes’.

“Supplement Table 1 lists LAMP Route names, NAAMP numbers, and the latitude and longitude of first stop. Figure 1 is a state map showing LAMP route starting points.”

3. “In your Methods section, please provide additional information regarding the permits you obtained for the work. Please ensure you have included the full name of the authority that approved the study sites access and, if no permits were required, a brief statement explaining why.”

Response: We rewrote the sentence to include more detail: ”All routes and stops were on public roads, and since volunteers only listened for frogs and did not either come into contact with them or lure them in, no permits were required for the surveys.”

4. We note that you have stated that you will provide repository information for your data at acceptance. Should your manuscript be accepted for publication, we will hold it until you provide the relevant accession numbers or DOIs necessary to access your data. If you wish to make changes to your Data Availability statement, please describe these changes in your cover letter and we will update your Data Availability statement to reflect the information you provide.

Response: We have been assigned a DOI address for the data release. The address will be active for data downloading by the end of January 2021. The citation has been added to the manuscript. It is: Carter J., Louisiana Amphibian Monitoring Program Survey Frog Call Observation Data: 1997-2017. U.S. Geological Survey data release https://doi.org/10.5066/P9VNBWM2.

5. “We note that Figure 1 in your submission contain map images which may be copyrighted.”

Response: The basemap is from ESRI online.  It is not copyrighted.  Most of the basemap is USGS DRG or topo data. The other elements in the map (the locations of LAMP routes, boarders between regions) were created by the authors working with a GIS professional. As a product of the government, it is not copyrighted.

**Comments from the reviewers**

Reviewer #1:

This study reviewed and analyzed frog call data from LAMP and evaluated the species richness, diversity and distributions, population dynamics of the three survey regions in Louisiana. The authors found the frog community of North region is in the middle between the South and Florida parishes. The authors also concluded monitoring for more than 10 years is vital to assess population and occupancy trends. For amphibians, 10 years are usually enough to evaluate the population dynamics. Therefore, my major concern is the survey method to count the abundance in this study. Has been it test the correlation between call indexes and species abundance? The frog call activities can largely fluctuate when ambient temperature or breeding stages (especially important for explosive breeding species) vary. Were these variables added to the statistic models? In addition, did these surveys complete at daytime or at night or both? I strongly suggest the authors…

RESPONSES: The reviewer asks several questions about our analyses and methods.

(1) Is there a correlation between call indexes and species abundance? Calling can be correlated with, but not a direct or precise measure of abundance. If you never hear a particular species calling in an area, the probability of it being present is very low. Likewise, if you hear a species calling frequently and at a high (3) value, you can assume it is abundant. In some cases however, a species may be an ‘explosive breeder’ whereby all the individuals in a given location call and breed at one time, usually when weather conditions align up just right. In those cases the call indexes will always be a 3, and in the example of the eastern narrow-mouthed toad. Since this kind of behavior varies significantly by species, we did not review the detailed natural history for every species covered in this manuscript. We felt that was beyond the scope of this manuscript.
(2) The impact of temperature and other weather conditions? Calling is very likely correlated with current and recent weather conditions and events, but, the weather data was not available for this set of analyses. It should be pointed out however, there are LAMP windows and survey protocols. These include seasonal minimum sampling temperatures, maximum wind speeds for sampling, etc. They are there in part to make certain that observations are made on comparable nights. In the future we’d like to take a closer look at the weather data.
(3) The time of day for the surveys and other protocol information. This information was in a table that was removed during our editing prior to submission. We did provide a reference for NAAMP protocols [reference 3, Weir and Mossman 2005]. We added more detail by modifying the first paragraph of the methods section.

“The LAMP followed the NAAMP survey protocols [3]. Each survey was initiated a ½ hour or later after sunset, and before midnight. The surveys were run three times a year during windows meant to capture ‘winter’, ‘spring’ or ‘summer’ calling frog species. All routes and stops were on public roads, and since volunteers only listened for frogs and did not either come into contact with them or lure them in, thus permits were not required for the surveys.”

Reviewer #2: Overall, most everything was fine with few errors that I caught:

line 130: "of" prior to "west" can be deleted.

Fixed

line 139: There's a strange placement of "nor" at the end of a sentence. "...did not need permits nor." Reword.

Fixed. See above for rewrite of that part of the methods section.

line 144: Just remove the miles and stick with km. No scientific article should be using miles.

Removed “miles”

line 157: I think your "(1)" is supposed to be an "(A)"

Fixed

line 263: "Were" not "Where"

Fixed

line 449: Remove "it" in "...of runs completed make it direct..."

Fixed

line 494: "where" not "were"

Fixed

line 519: sci name not in italics

Fixed

line 520: sci name not in italics

Fixed

Reviewer 2 wrote:

“Perhaps it is just me, but I had to read lines 155-156 a few times. Initially, I thought if condition B is satisfied, then A is automatically satisfied, in which case listing A is null. Eventually I got it. If other reviewers perhaps had a similar issue, needing to read this sentence a few times, then I'd suggest maybe providing an example. Or perhaps suggesting B first, and then A. "...routes had to have: (A) Surveys need to span 20 or more years, and (B) within that time span, there need to be data sets from 8 or more different years." I think it was the small (8) to large (20) that kind of threw me. So, I would suggest rewording to make the sentence/ conditions a little more clear, if other reviewers noted this area as being a bit awkward. If I'm the only one, then disregard.”

Response: I removed the (A), (B) conditions. They were intended to highlight the conditions needed to conduct certain kinds of analyses but seem to only confused the reader.

I rewrote as follows:

“The routes used depended on the analysis (Table 1). All stops with one or more species calling were used for analyses of species richness, phenology, and average call intensity (54 routes met these conditions). All stops with two or more species calling were used for co-occurrence analyses (45 routes met these conditions). For trend analysis using general linear models, routes had to have data sets from 8 or more different years, and the surveys needed to span 20 or more years (21 routes met these conditions). For General Additive Model (GAM) analysis route data had to have data sets from 8 or more different years, surveys spanning 20 or more years, and no gaps in the data of more than 5 years (19 routes met these conditions).”

**USGS Review Notes:** In response to requirements and suggestions made during our internal approval process, we made a few minor modifications. These were: clarifications and corrections on funding and affiliations; adding y-axis labels to the supplemental figures; and reworded sentences that could potentially be considered ‘prescriptive language’ directed at management agencies. ‘Prescriptive language’ is wording that says or suggests management or a government agency ‘should do something’. The two sentences flagged by the review process have been worded so that they are non-prescriptive.

I hope you find the revised manuscript acceptable.

Sincerely yours,
