# Supplementary material for: The Louisiana Amphibian Monitoring Program from 1997 to 2017: Results, analyses, and lessons learned
Source: PLoS One. 2021 Sep 30;16(9):e0257869. doi: 10.1371/journal.pone.0257869 (PMC8483421; doi:10.1371/journal.pone.0257869)
Supplement: Attachment — Submitted filename: PLOS Reconcillation June 28, 2021.doc [file pone.0257869.s004.doc]

United States Department of the Interior

U.S. GEOLOGICAL SURVEY

# Jacoby Carter, Ph.D.

Research Ecologist

Wetland and Aquatic Research Center

700 Cajundome Boulevard

Lafayette, Louisiana 70506

Cell: 3370781-4882

Phone: 337-266-8620

Fax: 337-266-8586

e-mail: jacoby_carter@usgs.gov

To:	Janice L. Bossart, Academic Editor

Date:	Monday, June 28, 2021

Re:	Reconciliation Letter for PONE-D-20-21124R1.

The Louisiana Amphibian Monitoring Program from 1997 to 2017: Results, Analyses, and Lessons Learned.

Dear Ms. Bossart,

I have revised the above manuscript as per your, and the reviewer’s comments. I appreciate the extra time you gave me (July 2) to complete my revisions and response.

Please find below the message that was sent May 5 with comments as to what changes or revisions were made in response, or the reasons why the changes were not made. This

In order to make certain I didn’t miss any comments/suggestions/changes asked, I have copied the letter I was sent on May 5, 2021 below. Comments are followed by responses in Times New Roman.

I hope you find these changes acceptable and sufficent.

Sincerely yours,

Jacoby

-------------------------------------------------------------------------------------------------------------------

From: em.pone.0.731669.1f6fe84e@editorialmanager.com <em.pone.0.731669.1f6fe84e@editorialmanager.com> on behalf of PLOS ONE <em@editorialmanager.com>
Sent: Wednesday, May 5, 2021 2:47 AM
To: Carter, Jacoby <carterj@usgs.gov>
Subject: [EXTERNAL] PLOS ONE Decision: Revision required [PONE-D-20-21124R1] - [EMID:780cb4c241cff8f2]

----- PONE-D-20-21124R1
The Louisiana Amphibian Monitoring Program from 1997 to 2017: Results, Analyses, and Lessons Learned.
PLOS ONE

Dear Dr. Carter,

Thank you for submitting your manuscript to PLOS ONE. After careful consideration, we feel that it has merit but does not fully meet PLOS ONE’s publication criteria as it currently stands. Therefore, we invite you to submit a revised version of the manuscript that addresses the points raised during the review process.

Although I'm keeping the editorial decision as Minor Revision (the data and analyses are largely solid), the Introduction and Discussion, nonetheless, need some solid reworking.  As reviewer 3 noted, neither of these sections are well-developed.  I fully agree.  PLoS ONE is an international journal and the Introduction needs to place the study into some broader context rather than simply describing a program specific to a single state in the United States.

For example, what was the motivation for the study?  In what way will the results be relevant?

Response: Introduction. I rearranged the sentences in the introduction to address this problem as to ‘Why?’ of the study. While it was there in the first sentence, (perceived amphibian declines), it got lost in the details that followed. I also rewrote a few sentences to clarify which questions we were answering with LAMP data. Finally, I removed the reference to a previous study in the introduction. The reference to the previous study broke the flow of the introduction and really didn’t add to the set up for the Introduction.

Discussion Structure. Here we focused on the community level and species level questions. There were many different analytical approaches we used with the LAMP data to answer the multiple questions. In some cases more that one approach was used to answer a question.

We used the discussion to tie what was presented in the Results section directly back to the questions we asked at the end of the Introduction. To make this section cleaner, after restating the question, I provide a direct, simple answer. I then follow up with a paragraph that abstracts from the results. I removed as detailed recapitulation from the results that I thought I could.

Conclusion. The conclusion section now focuses on the three broad questions. “Can this method be used to monitor for changes in frog abundance or distribution?” “Did we see changes in abundance or frog distributions?” “Are their regional differences in frog distribution and abundance?” Followed by a section on why continued monitoring is important.

Additional Changes-

• In order to distinguish between average call index (ACI) for all species in a given region/route/run combination and those for just one species in a given region/route/run combination I now use ‘ACI’ when referring to all species on a given region/route/run and ‘sACI’ when referring to just one species on a given region/route/run. This should remove any ambiguity.

Hutto Jr, D., & Barrett, K. (2021). Do urban open spaces provide refugia for frogs in urban environments?. *PloS one*, *16*(1), e0244932.

Rivas, G. A., et al. (2021). Biogeographical patterns of amphibians and reptiles in the northernmost coastal montane complex of South America. *PloS one*, *16*(3), e0246829.

Ceron, K., Santana, D. J., & Valente-Neto, F. (2020). Seasonal patterns of ecological uniqueness of anuran metacommunities along different ecoregions in Western Brazil. *PloS one*, *15*(9), e0239874.

All reviewers have had favorable comments about the overall quality of your data and statistical approaches used.  I look forward to receiving a revised version, where the Introduction and Discussion and have been better fleshed out.

Thank you for those references.

Please submit your revised manuscript by Jun 19 2021 11:59PM. If you will need more time than this to complete your revisions, please reply to this message or contact the journal office at plosone@plos.org. When you're ready to submit your revision, log on to https://www.editorialmanager.com/pone/ and select the 'Submissions Needing Revision' folder to locate your manuscript file.

Please include the following items when submitting your revised manuscript:

- A rebuttal letter that responds to each point raised by the academic editor and reviewer(s). You should upload this letter as a separate file labeled 'Response to Reviewers'.
- A marked-up copy of your manuscript that highlights changes made to the original version. You should upload this as a separate file labeled 'Revised Manuscript with Track Changes'.
- An unmarked version of your revised paper without tracked changes. You should upload this as a separate file labeled 'Manuscript'.

If you would like to make changes to your financial disclosure, please include your updated statement in your cover letter. Guidelines for resubmitting your figure files are available below the reviewer comments at the end of this letter.

If applicable, we recommend that you deposit your laboratory protocols in protocols.io to enhance the reproducibility of your results. Protocols.io assigns your protocol its own identifier (DOI) so that it can be cited independently in the future. For instructions see: [http://journals.plos.org/plosone/s/submission-guidelines#loc-laboratory-protocols](https://gcc02.safelinks.protection.outlook.com/?url=http%3A%2F%2Fjournals.plos.org%2Fplosone%2Fs%2Fsubmission-guidelines%23loc-laboratory-protocols&data=04|01|carterj@usgs.gov|c810898f12684d80487a08d932a53a87|0693b5ba4b184d7b9341f32f400a5494|0|0|637596507507459225|Unknown|TWFpbGZsb3d8eyJWIjoiMC4wLjAwMDAiLCJQIjoiV2luMzIiLCJBTiI6Ik1haWwiLCJXVCI6Mn0%3D|2000&sdata=DiwbgHUfiUOMqwqlVAeDnv%2BOOJaBnLRwQiWFoaE%2B9Qw%3D&reserved=0). Additionally, PLOS ONE offers an option for publishing peer-reviewed Lab Protocol articles, which describe protocols hosted on protocols.io. Read more information on sharing protocols at [https://plos.org/protocols?utm_medium=editorial-email&utm_source=authorletters&utm_campaign=protocols](https://gcc02.safelinks.protection.outlook.com/?url=https%3A%2F%2Fplos.org%2Fprotocols%3Futm_medium%3Deditorial-email%26utm_source%3Dauthorletters%26utm_campaign%3Dprotocols&data=04|01|carterj@usgs.gov|c810898f12684d80487a08d932a53a87|0693b5ba4b184d7b9341f32f400a5494|0|0|637596507507459225|Unknown|TWFpbGZsb3d8eyJWIjoiMC4wLjAwMDAiLCJQIjoiV2luMzIiLCJBTiI6Ik1haWwiLCJXVCI6Mn0%3D|2000&sdata=dkrHZa6zfQth0tP0FzmtDdQtmTLtWQ1LJwReA3x3DiA%3D&reserved=0).

We look forward to receiving your revised manuscript.

Kind regards,

Janice L. Bossart
Academic Editor
PLOS ONE

Journal Requirements:

Please review your reference list to ensure that it is complete and correct. If you have cited papers that have been retracted, please include the rationale for doing so in the manuscript text, or remove these references and replace them with relevant current references. Any changes to the reference list should be mentioned in the rebuttal letter that accompanies your revised manuscript. If you need to cite a retracted article, indicate the article’s retracted status in the References list and also include a citation and full reference for the retraction notice.

Additional Editor Comments (if provided):


[Note: HTML markup is below. Please do not edit.]

Reviewers' comments:

Reviewer's Responses to Questions

Comments to the Author

1. If the authors have adequately addressed your comments raised in a previous round of review and you feel that this manuscript is now acceptable for publication, you may indicate that here to bypass the “Comments to the Author” section, enter your conflict of interest statement in the “Confidential to Editor” section, and submit your "Accept" recommendation.

Reviewer #2: All comments have been addressed

Reviewer #3: (No Response) Did not review earlier drafts. Is a new reviewer.

2. Is the manuscript technically sound, and do the data support the conclusions?

The manuscript must describe a technically sound piece of scientific research with data that supports the conclusions. Experiments must have been conducted rigorously, with appropriate controls, replication, and sample sizes. The conclusions must be drawn appropriately based on the data presented.

Reviewer #2: Yes

Reviewer #3: Yes

3. Has the statistical analysis been performed appropriately and rigorously?

Reviewer #2: Yes

Reviewer #3: Yes

4. Have the authors made all data underlying the findings in their manuscript fully available?

The [PLOS Data policy](https://gcc02.safelinks.protection.outlook.com/?url=http%3A%2F%2Fwww.plosone.org%2Fstatic%2Fpolicies.action%23sharing&data=04|01|carterj@usgs.gov|c810898f12684d80487a08d932a53a87|0693b5ba4b184d7b9341f32f400a5494|0|0|637596507507578701|Unknown|TWFpbGZsb3d8eyJWIjoiMC4wLjAwMDAiLCJQIjoiV2luMzIiLCJBTiI6Ik1haWwiLCJXVCI6Mn0%3D|2000&sdata=IzRvO4o7jmUpKZY%2BegtLvBuxomDWn2j%2FeS2z530TA0Y%3D&reserved=0) requires authors to make all data underlying the findings described in their manuscript fully available without restriction, with rare exception (please refer to the Data Availability Statement in the manuscript PDF file). The data should be provided as part of the manuscript or its supporting information, or deposited to a public repository. For example, in addition to summary statistics, the data points behind means, medians and variance measures should be available. If there are restrictions on publicly sharing data—e.g. participant privacy or use of data from a third party—those must be specified.

Reviewer #2: Yes

Reviewer #3: Yes

5. Is the manuscript presented in an intelligible fashion and written in standard English?

PLOS ONE does not copyedit accepted manuscripts, so the language in submitted articles must be clear, correct, and unambiguous. Any typographical or grammatical errors should be corrected at revision, so please note any specific errors here.

Reviewer #2: Yes

Reviewer #3: Yes

6. Review Comments to the Author

Please use the space provided to explain your answers to the questions above. You may also include additional comments for the author, including concerns about dual publication, research ethics, or publication ethics. (Please upload your review as an attachment if it exceeds 20,000 characters)

Reviewer #2: As I did not have many issues with the first draft, and other reviewers had more - this version is more than acceptable to me.

Reviewer #3: My overall impression is that I am impressed with this massive project and the data. Very important that they are presented. The analysis are appropriate and are communicated clearly.

I would wish for more of a proper discussion section. Except for ll. 542-550, the current version just reads as a rehash of the Results. Even though I personally don’t find call surveys my cup of tea, this is such a large effort and such a large data set, that any mechanical problems that would usually be inherent in a report like this one are simply overrun by the enormity of the data. So, I’m a hater, but I love the data and effort reported in this manuscript and hope to read it in print soon.

I have rewritten the discussion so it is more succint and minimizes repeat of what is in the Results section. I appreciate the author’s honesty about his ‘love’ for call survey data.

(This is my first view of this manuscript; many of the parts read like they are responses to previous reviewers and so feel a little clunky. I've mentioned these points below).

Other comments intended to help improve the writing:

Can the volunteers willing to be named be included? This huge project is a great opportunity to recognize them.

This was discussed by the team and some of the current vollunters. There was a mutual agreement not to name them in either the database we uploaded or the the acknowledgement.

31 species? There are 32 reported species in Louisiana.

It depends on how you count. Boundy and Carr (2017) ‘Amphibians and Reptiles of Louisiana’ has accounts for 33 frog and toad species, but two pairs of species (Fowler’s & East Texas Toads, and Blanchard’s & Eastern Cricket Frogs) can be distinguished only using genetic data and their respective distributions within the state are unclear. So for our analysis purposes the pairs are treated as the same species (that is, Fowler’s Toad and Eastern Cricket Frog). Frog taxonomy has seen radical revisions over the last 20 years due to genetic ‘insights’. What was ‘*Rana*’ became ‘*Lithobates*’ and for some, back to ‘*Rana*’ again or others. Something similar happened with toads (‘*Bufo’* / ‘*Anaxyrus’*) and treefrogs (‘*Hyla*’ / ‘*Dryophytes*’). So I prefer stick to the 31 species we refer to in the manuscript.

l. 55 - ‘synchronicity’ is unclear

Rewrote.

Make the abstract a single paragraph.

Done.

l. 64 - remove ‘perceived’ and ‘North American’

I will leave those words in. First, ‘perceived’. One of the reasons these surveys were established was to determine if the ‘declines’ where real. There was (and is) concern that the ‘declines’ might have been due to observer bias. For example a researcher picks a place to study amphibians because s/he knows lots of amphibians are there. Over time, natural population cycles may lead to a decline in numbers of the studied population. If this happens with lots of researchers, they may have a sense that populations are declining everywhere when they are not. The random observations points and long term monitoring of the NAAMP program were an attempt to get around this bias. Our results support this suspicion.

Second, ‘North America’, while amphibian declines are a globally noted phenomena NAAMP was designed to address that question as it applies to North America in general and the US in particular.

Is this really an Introduction? It reads as a description of the project. It is not placed in the context of the global question of amphibian decline and the monitoring programs put in place to address this problem.

True, we didn’t place it in the context of the global issue. We focused on the North American aspect. The question is “Are the observed declines ‘real’?” Our the focus in this study, are there declines in Louisiana? We removed details on how the survey was conducted.

ll. 69-72 and 77-79 are better placed in Methods.

I removed details on how the survey is conducted. They are already covered in the methods section.

l. 80. move [1] before period.

Thanks for catching that. I was fixed during the revision process. Additionally, I did a global search for other instances of “. [“ and found none that were inappropriate.

ll. 92-93 - this is not a novel question; species co-occurences and associated phenology and habitat are already known and described in the literature. And this study is not the best way to address this.
ll. 98-99 - see previous.

Perhaps there might be better ways to address those questions. Perhaps those questions have been asked before. But those questions have never been addressed comprehensively for Louisiana, or with this kind of data over such a large geographical range (the entire state of Louisiana), over this long a period of time (20 years). Since we have locations and observations, it would be a crime (and a complete waste of data) not to use it to explore those questions.

ll. 113-116 and l. 119 - The Florida Parishes make sense because it’s a biogeographic thing. But do better at explaining the choice of 31 degrees. I think it’s a simply reason, but write it. At this point, it reads as something arbitrary but I doubt that it was chosen arbitrarily.

Thanks. I provided an explanation and reference in methods.

“This line reflects a geographical break in the randomly assigned route locations between north and south Louisiana; and the 16°C average annual temperature line [4], were north of that line the winters are colder, longer, and frogs start calling later in the year.”

TABLE 1 - I understand why there might be a wide range of variation in number of stops. But try to describe why.

It was in a different location of the paper but I moved it. Under the heading Survey Routes in the methods section I added:

“The total number of stops observed varied between routes for two reasons. Not all routes were surveyed every year, or completely surveyed in a given year. “

l. 161 (and elsewhere) - use your term ‘observers’ not ‘surveyors’

I changed all instances of ‘surveyors’ to ‘observers’

l.177 - data ‘are’; replace ‘is’

done

l. 181 - see previous

done

l.209-221 - remove the descriptions of species indices calculations; these are common knowledge.

How ‘common’ this knowledge is depends on who you are. I have met many biologists at all levels of training, who were not trained in community ecology indexes. It doesn’t take up more than a couple Sentences and a few equations, and knowing what they are is crucial to interpreting the analyses that follow. Also we use the indexes for two differen things. First we use it for as a species diversity measure. However, we also use the ‘J’ index to compare sample effort between regions. To explain how the ‘J’ index (something many biologists regard as a ‘species’ index) might be applied to measuring sampling effort, it is worth reviewing what the ‘J’ statistic actually means. We prefer to leave it in to prevent confusion as to how we are using the index.

The GAM is description is critical to this manuscript and it’s clear. Nice job!

Thanks.

ll. 244-248 and Fig. 2. - this section feels like something that may have been requested by an earlier reviewer. I find it unnecessary; it’s intuitive and use of the GAM analyses are fairly common. A bit like explaining how a regression works.

Again, as in the previous comment (l.209-221), I have found knowledge of statistical tests uneven across the field of herpetology. It doesn’t cost much in terms of space to explain what we are doing in general terms and to help the reader follow what comes next. As for the pattern classification of the GAMs, that is not as commonly done and a picture is worth a thousand words.

l. 261-262 - I’m interested! Why were there fewer routes?

For a variety or reasons we have had declines in volunteer participation. However, I’d say a major reason we saw a sharp decline after that year was that volunteers were informed that the USGS was shutting down the NAAMP and they were discouraged. I didn’t mention it because this USGS approved manuscript is not the place to discuss that issue.

l. 268 - I LOVE the numbers 54 and (especially) 12,792! This line represents how much work has been put into this project and why I think some place for the volunteers names would improve this manuscript.

This was a matter of some discussion by the authors and volunteers. One problem was that there were many observers that had left the program and in some cases their routes were taken up by others, in other cases not. Contacting past volunteers for permission to use their names has been difficult. Secondly, there was the issue that some observers had 100’s of stops to their credit and others only a few dozen. While we recognized these ‘champion observers’ during our annual meetings, we didn’t know how to do this in a very long acknowledgement section. Pre-Covid (as late as December 2019) we would hold annual meetings of all the volunteers at the beginning of December, a month before the start of the survey season in the south. During those meetings we discussed the progress we were making on this analyses, and updated them on any pertinent rules and regulations, and shared stories from the previous seasons’ surveys. Few were interested in acknowledgement in this manuscript for their volunteer work. However, all of the observers were in agreement about one thing. They wanted this data published so their hard work could be collectively recognized. The best thing we can do to honor their work is to get this in press! When published, we will provide the link to the published article to as many of the volunteers as I can contact in a thank you email.

l.272 - 11 species calling at a single stop! That is neat and impressive and shows how good the observers were.

I agree.

l. 278-284 - I don’t understand the reason to include this.

It demonstrates that for some species call intensity is not a good measure of abundance and thus can not be used across the board. Many already know this, but some who may be reading this may not. This data demonstrates it for some Louisiana frogs and toads. It was a facile example, but why not put it in there to make the point?

Fig. 5 - This could be deleted and the regression statistics simply incorporated into the text.

The figure works better at demonstrating the regression fit and scatter across the entire observation range. I’d prefer to keep it. Also, as a dyslexic, I (Jacoby) am more of a visual thinker. The graph gives me a better idea of what is going on. Some of our readers may be similarly inclined.

ll. 306-314 and elsewhere - Taxonomy matters in some journals and I’ll assume that it does in PLOS ONE. It is not “spring peepers”; it is Spring Peepers; it is not “Southern (Acris gryllus) and northern cricket frogs (Acris crepitans)”; it is Southern Cricket Frogs (Acris gryllus) and Northern Cricket Frogs (Acris crepitans).

Thanks. We capitalized the first letter of all common names.

In addition, at present, the preferred use is Hyla not Dryophytes (along with the associated gender changes in species epithets. See Crother et al for current taxonomy.

Current USGS guidance requires that we follow ITIS (itis.gov) as our standard for taxonomic names. In truth I prefer *Hyla* as well. But internal approval requires we use what’s in ITIS (which considers *Hyla* to be invalid) in all USGS funded publications. To clarify the taxonomy, I (Jacoby) have indicated that we are using ITIS as our nomenclature standard in the body of the text.

ll. 319-322 - I think this is an excellent place for proper digging. Why not compare to older patterns? For example, compare with Fig. 6 in Dundee and Rossman. This would allow one to address the question of changing call phenology associated with a 35 year change in climate.

Dr. Beachy, don’t imagine we were not tempted. This manuscript (focused on analyses of data from the LAMP surveys and is already large) is not the best place to do a tangential deep dive into comparisons with historical accounts. If you’re interested in doing a comparison with historical accounts, I (Jacoby) suggest you partner with Jeff Boundy. Jeff has very detailed knowledge of frog and toad distributions, and phenology going back 20+ years. Jeff has also been working with the state’s natural history museum for just as long.

7. PLOS authors have the option to publish the peer review history of their article ([what does this mean?](https://gcc02.safelinks.protection.outlook.com/?url=https%3A%2F%2Fjournals.plos.org%2Fplosone%2Fs%2Feditorial-and-peer-review-process%23loc-peer-review-history&data=04|01|carterj@usgs.gov|c810898f12684d80487a08d932a53a87|0693b5ba4b184d7b9341f32f400a5494|0|0|637596507507578701|Unknown|TWFpbGZsb3d8eyJWIjoiMC4wLjAwMDAiLCJQIjoiV2luMzIiLCJBTiI6Ik1haWwiLCJXVCI6Mn0%3D|2000&sdata=RIqoOHZoA%2Be96qBCQoGeanVa4tpIQ9aBrCBo9ypgBoU%3D&reserved=0)). If published, this will include your full peer review and any attached files.


If you choose “no”, your identity will remain anonymous but your review may still be made public.


Do you want your identity to be public for this peer review? For information about this choice, including consent withdrawal, please see our [Privacy Policy](https://gcc02.safelinks.protection.outlook.com/?url=https%3A%2F%2Fwww.plos.org%2Fprivacy-policy&data=04|01|carterj@usgs.gov|c810898f12684d80487a08d932a53a87|0693b5ba4b184d7b9341f32f400a5494|0|0|637596507507588664|Unknown|TWFpbGZsb3d8eyJWIjoiMC4wLjAwMDAiLCJQIjoiV2luMzIiLCJBTiI6Ik1haWwiLCJXVCI6Mn0%3D|2000&sdata=ox2E4LqLvwKUzCfBRebcHezmB%2FIg5br2T4cbDLqaS0o%3D&reserved=0).

Reviewer #2: No

Reviewer #3: Yes: Christopher K. Beachy

I would like to thank all the reviewers. Dr. Beachy’s comments and suggestions, as well as that of the editor, in this round of revision were much appreciated. Some of my responses were directed to Dr. Beachy directly. I don’t mind if they are forwarded to him. I appreciated his insights and comments.

[NOTE: If reviewer comments were submitted as an attachment file, they will be attached to this email and accessible via the submission site. Please log into your account, locate the manuscript record, and check for the action link "View Attachments". If this link does not appear, there are no attachment files.]

While revising your submission, please upload your figure files to the Preflight Analysis and Conversion Engine (PACE) digital diagnostic tool, [https://pacev2.apexcovantage.com/](https://gcc02.safelinks.protection.outlook.com/?url=https%3A%2F%2Fpacev2.apexcovantage.com%2F&data=04|01|carterj@usgs.gov|c810898f12684d80487a08d932a53a87|0693b5ba4b184d7b9341f32f400a5494|0|0|637596507507588664|Unknown|TWFpbGZsb3d8eyJWIjoiMC4wLjAwMDAiLCJQIjoiV2luMzIiLCJBTiI6Ik1haWwiLCJXVCI6Mn0%3D|2000&sdata=deDxnPde%2BaprGhatEn8HEHhaDZYpGJDaJopqFAMOub8%3D&reserved=0). PACE helps ensure that figures meet PLOS requirements. To use PACE, you must first register as a user. Registration is free. Then, login and navigate to the UPLOAD tab, where you will find detailed instructions on how to use the tool. If you encounter any issues or have any questions when using PACE, please email PLOS at [figures@plos.org](mailto:figures@plos.org). Please note that Supporting Information files do not need this step.

*In compliance with data protection regulations, you may request that we remove your personal registration details at any time.* [*(Remove my information/details)*](https://gcc02.safelinks.protection.outlook.com/?url=https%3A%2F%2Fwww.editorialmanager.com%2Fpone%2Flogin.asp%3Fa%3Dr&data=04|01|carterj@usgs.gov|c810898f12684d80487a08d932a53a87|0693b5ba4b184d7b9341f32f400a5494|0|0|637596507507598616|Unknown|TWFpbGZsb3d8eyJWIjoiMC4wLjAwMDAiLCJQIjoiV2luMzIiLCJBTiI6Ik1haWwiLCJXVCI6Mn0%3D|2000&sdata=36xsAW4Y3nA4P4nl7fbiGFwFfN8lOkZskUya3lwSif0%3D&reserved=0)*. Please contact the publication office if you have any questions.*
