# Supplementary material for: The Louisiana Amphibian Monitoring Program from 1997 to 2017: Results, analyses, and lessons learned
Source: PLoS One. 2021 Sep 30;16(9):e0257869. doi: 10.1371/journal.pone.0257869 (PMC8483421; doi:10.1371/journal.pone.0257869)
Supplement: Attachment — Submitted filename: PLOS Cover Letter and Reconcil 07292021.doc [file pone.0257869.s006.doc]

United States Department of the Interior

U.S. GEOLOGICAL SURVEY

# Jacoby Carter, Ph.D.

Research Ecologist

Wetland and Aquatic Research Center

700 Cajundome Boulevard

Lafayette, Louisiana 70506

Cell: 3370781-4882

Phone: 337-266-8620

Fax: 337-266-8586

e-mail: jacoby_carter@usgs.gov

**To:	Janice L. Bossart, Academic Editor**

**Date:	Thursday 29, 2021**

**Re:**	Reconciliation Letter for PONE-D-20-21124R1.

The Louisiana Amphibian Monitoring Program from 1997 to 2017: Results, Analyses, and Lessons Learned.

Dear Dr. Bossart,

You wrote:

“Dear Dr. Carter,

Thank you for submitting your manuscript to PLOS ONE. After careful consideration, we feel that it has merit but does not fully meet PLOS ONE’s publication criteria as it currently stands. Therefore, we invite you to submit a revised version of the manuscript that addresses the points raised during the review process.

I'm sorry I need to send the manuscript back to you again, but PLoS One manuscripts are not copy edited.  Once I accept it will go to the production staff.  Just a few things:

- Line 118 (Clean copy), i.e. Table 1 footnote.  Although you indicated 'was' had been changed to 'were', it hasn't.  Please change, '...data from that route were...'
- Table 1.  Please ensure the entire Table can be see in Draft view in Word since it extends past the right margin.  I can't doublecheck as I only receive a pdf.
- Scientific nomenclature.  I appreciate your effort, but genus is generally only spelled out on first use in the main text and at the start of a sentence and abbreviated otherwise.  Sorry, I should have caught this issue earlier, but it only hit me when even more were spelled out in this version.  In the main text, spell out genus once and thereafter abbreviate.  For example, spell out Lithobates on line 276, but otherwise only use the abbreviation for any Lithobates species unless it starts a sentence.  Ditto for all other genera.  In many spots where genus is currently spelled out fully, you'll need to abbreviate.  Don't change how you currently have nomenclature in the abstract, any of the tables, or the figure 8 caption.
- Line 479-480 (Clean copy).  Delete 'the' before each species (re-abbreviate genus as necessary).

Please submit your revised manuscript by Sep 12 2021 11:59PM. If you will need more time than this to complete your revisions, please reply to this message or contact the journal office at plosone@plos.org. When you're ready to submit your revision, log on to https://www.editorialmanager.com/pone/ and select the 'Submissions Needing Revision' folder to locate your manuscript file.”

RESPONSE

I have made the following changes.

1 & 2 I corrected the footnote on line 118. I checked and in Draft View the entire table is visible. Thanks for asking me to check.

3. Okay, I get it now. First use, genus and species. Second use Initial of genus and species, unless the Genus is the first word in the sentence. I went through the manuscript using search feature for the first instance of the full genus-species name and then afterwards to conform. As an additional change, to be consistent, in the few cases where I used the common name after its first introduction I switched to the Latin binomal.

4. I deleted ‘the’ before the species names in Lines 479-480. The ‘the’ was left over when I changed from ‘…the CommonNameOfSpecies (*Latin binomial*)…’ to, ‘….*L. binomial*….’ I reviewed the document and found one other instance where that happened and fixed it.
